# IL-33 activates group 2 innate lymphoid cell expansion and modulates endometriosis

**DOI:** 10.1172/jci.insight.149699

**Published:** 2021-12-08

**Authors:** Jessica E. Miller, Harshavardhan Lingegowda, Lindsey K. Symons, Olga Bougie, Steven L. Young, Bruce A. Lessey, Madhuri Koti, Chandrakant Tayade

**Affiliations:** 1 Department of Biomedical and Molecular Sciences, Queen’s University, Kingston, Ontario, Canada.; 2 Department of Obstetrics and Gynecology, Kingston General Hospital, Kingston, Ontario, Canada.; 3 Department of Obstetrics and Gynecology University of North Carolina, Chapel Hill, North Carolina, USA.; 4 Department of Obstetrics and Gynecology, Wake Forest Baptist Health, Winston-Salem, North Carolina, USA.

**Keywords:** Immunology, Reproductive Biology, Cellular immune response, Mouse models, Th2 response

## Abstract

Chronic inflammation and localized alterations in immune cell function are suspected to contribute to the progression of endometriosis and its associated symptoms. In particular, the alarmin IL-33 is elevated in the plasma, peritoneal fluid, and endometriotic lesions from patients with endometriosis; however, the exact role of IL-33 in the pathophysiology of endometriosis is not well understood. In this study, we demonstrate, in both humans and a murine model, that IL-33 contributes to the expansion of group 2 innate lymphoid cells (ILC2s), and this IL-33–induced ILC2 expansion modulates the endometriosis lesion microenvironment. Importantly, we show that IL-33 drives hallmarks of severe endometriosis, including elevated inflammation, lesion proliferation, and fibrosis, and that this IL-33–induced aggravation is mediated by ILC2s. Finally, we demonstrate the functionality of IL-33 neutralization as a promising and potentially novel therapeutic avenue for treating the debilitating symptoms of endometriosis.

## Introduction

Approximately 176 million women worldwide suffer from endometriosis. Together with the emerging associative risk of developing ovarian cancer (1) and the enormous economic burden (2, 3), endometriosis is a critical public health concern that is conspicuously understudied. Sampson’s widely accepted theory of pathogenesis suggests that during menstruation, shed endometrial fragments are refluxed into the peritoneal cavity where they evade immune surveillance and adhere to pelvic structures (4). These fragments then develop into endometrial-like lesions and subsequently exhibit chronic inflammation, undergo neuroangiogenesis, and become highly fibrotic (5). Patients experience an average diagnostic delay of 6–11 years (6), and even after procuring a diagnosis, their debilitating chronic pelvic pain, severe dysmenorrhea (painful periods), and infertility are often ineffectively managed by the current treatments.

In the healthy endometrium and peritoneum, a precise but dynamic immune balance is maintained (7, 8). However, in patients with endometriosis, the endometrium exhibits vast immune alterations and endometriotic lesions exhibit even greater immune deviations. Various dysfunctional immune pathways have been identified through large-scale transcriptomic analyses, cytokines/chemokine analyses, and immune cell profiling (reviewed extensively in refs. 9, 10). It has been postulated that immune dysfunction, including elevated alternatively activated (M2) macrophages, Tregs, Th2 cells, and elevated type 2 cytokines, facilitates the adhesion, invasion, angiogenesis, and fibrosis observed in the endometriotic lesion (9, 11). Additionally, the two most predominant symptoms of endometriosis, chronic pelvic pain and infertility, are often connected to aberrant inflammation (12, 13). However, the precise mechanisms that underpin how inflammation contributes to chronic pelvic pain and infertility are not well understood.

IL-33 is a member of the IL-1 cytokine family, and it exerts type 2 immune responses through its primary receptor, ST2 (14). Our research group and others have demonstrated that IL-33 is elevated in the peritoneal fluid (PF) and endometriotic lesion tissue of patients with deep infiltrating endometriosis and endometriomas (15–18). A large-scale genome-wide association study has identified a statistically significant single nucleotide polymorphism in the region of the IL-33 gene, suggesting that IL-33 dysfunction in endometriosis may be genetic in origin (19). Interestingly, IL-33 is a double-edged sword that can be protective or pathological depending on the tissue of origin and environmental niche (20). How the source of IL-33 contributes to its downstream function is at the forefront of the IL-33 biology field and is not well understood (21). However, we do know that in some tissues, IL-33 can form pathological niches with other cell types. For example, while astrocyte-derived IL-33 is critical for healthy neuronal development (22), stromal cell–derived IL-33 in the lung provides a pathologic niche, which exacerbates asthma, allergic responses, and helminth infections (23). Similarly, in adipose tissue, stromal cell–derived IL-33 stimulates the release of pathological type 2 immune cytokines, which impair visceral adipose tissue homeostasis and contribute to type 2 diabetes mellitus. We also know that IL-33 exerts its effects through various immune and nonimmune cells; however, many studies have shown that IL-33 primarily activates group 2 innate lymphoid cells (ILC2s) to perpetuate autoimmune and chronic inflammatory diseases (24). Using our murine model of endometriosis, we previously demonstrated that IL-33 likely has a pathological role in the progression of endometriosis, as it stimulated systemic inflammation and contributed to lesion proliferation (17). This pathological theory was validated by other research groups, which showed that IL-33 immunostaining was correlated with a greater extent of fibrosis (25) and both IL-33–deficient and ST2-deficient mice had significantly reduced lesion proliferation (26). Therefore, elevated levels of IL-33 are associated with severe endometriosis pathology, and IL-33 has been suggested as a promising therapeutic target; however, the molecular mechanisms and associated immune cells that underpin how IL-33 contributes to endometriosis are not understood.

In this study, we used samples from patients with endometriosis to understand the spatial arrangement of IL-33 in the endometriotic lesion, and for the first time to our knowledge, we identify the presence of ILC2s in the PF from patients with endometriosis. Using our well-established syngeneic allograft murine model of endometriosis, we demonstrate that exogenous peritoneal IL-33 administration led to hallmarks of severe endometriosis: excess peritoneal inflammation, immune cell recruitment, lesion proliferation, lesion neurogenesis, and lesion collagen deposition. Using two different methodologies, *RAG2–/–* mice treated with an anti-CD90.2 antibody (αCD90.2) (ILC2-depleted) and *RAG2^–/–^IL-2r*γ^*–/–*^ (ILC2-deficient) mice, we show that the IL-33–driven inflammation, immune cell recruitment, lesion proliferation, and fibrosis are in fact dependent on ILC2s. Our findings demonstrate the pathological role of IL-33–activated ILC2s in endometriosis, and we suggest that this axis could be a novel and promising nonhormonal therapeutic target for endometriosis.

## Results

### Human ILC2s are detected in the PF of patients with endometriosis.

**Figure 1:**
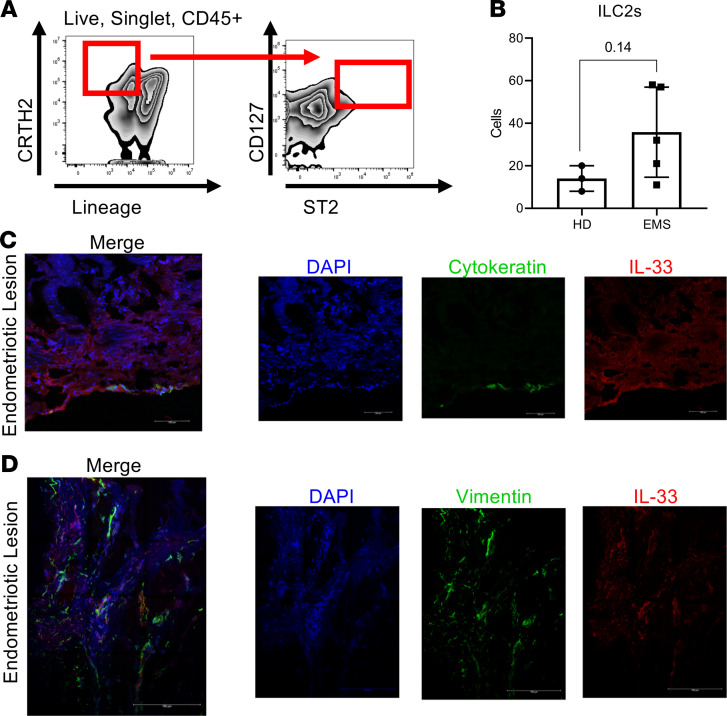
ILC2 abundance and IL-33 expression in samples from patients with endometriosis. (**A**) Representative flow cytometric analysis of group 2 innate lymphoid cells (ILC2s) in peritoneal fluid of healthy donors (HD) and patients with endometriosis (EMS). (**B**) The number of ILC2s in EMS (*n* = 5) and HD (*n* = 3). The *P* value (*P* = 0.14) for this comparison is shown. (**C** and **D**) Representative images of immunofluorescence staining demonstrating colocalization of IL-33 to epithelial cells (cytokeratin^+^) and to stromal cells (vimentin^+^), respectfully, in the endometriotic lesion microenvironment (*n* = 3). Scale bars: 100 μm (first image, **C** and **D**); 150 μm (second, third, and fourth image, **C** and **D**). Mean ± SD. Nonparametric Student’s *t* test with Mann-Whitney.

In other chronic inflammatory conditions, such as asthma, type 2 diabetes, and atopic dermatitis, elevated levels of IL-33 activate ILC2s to perpetuate type 2 inflammation and aggravate the symptoms of the disease (27–29). Elevated levels of IL-33 in PF from severe endometriosis cases is well established (15); however, for the first time to our knowledge, we report that live CD45^+^Lin^–^CD127^+^CD294^+^ST2^+^ cells (ILC2s) were detected in the PF from patients with endometriosis (Figure 1A and Supplemental Figure 1, A–F; supplemental material available online with this article; https://doi.org/10.1172/jci.insight.149699DS1). In fact, ILC2s in the PF from patients with endometriosis (*n* = 5) were more abundant compared with healthy fertile controls (*n* = 3), although the increase did not reach significance (*P* = 0.14) (Figure 1B).

### IL-33 colocalizes around epithelial and stromal endometriotic cells.

The endometriotic lesion is composed largely of epithelial, stromal, endothelial, and immune cells. As we previously reported, IL-33 is elevated in the endometriotic lesions compared with the patient’s matched endometrium and compared with the endometrium of healthy fertile controls (17). However, because IL-33 can act as either protective or pathological depending on the environmental niche, we sought to understand the endometriotic lesion microenvironment in greater detail. Using endometriotic lesions obtained from patients with severe endometriosis (*n* = 3), dual staining for IL-33 with cytokeratin (epithelial cell marker) (Figure 1C and Supplemental Figure 2, A–C) and vimentin (stromal cell marker) (Figure 1D and Supplemental Figure 2, D–F) revealed that IL-33 colocalizes with both stromal and epithelial cells in the lesion microenvironment.

### IL-33 stimulates inflammation in the local lesion microenvironment.

**Figure 2:**
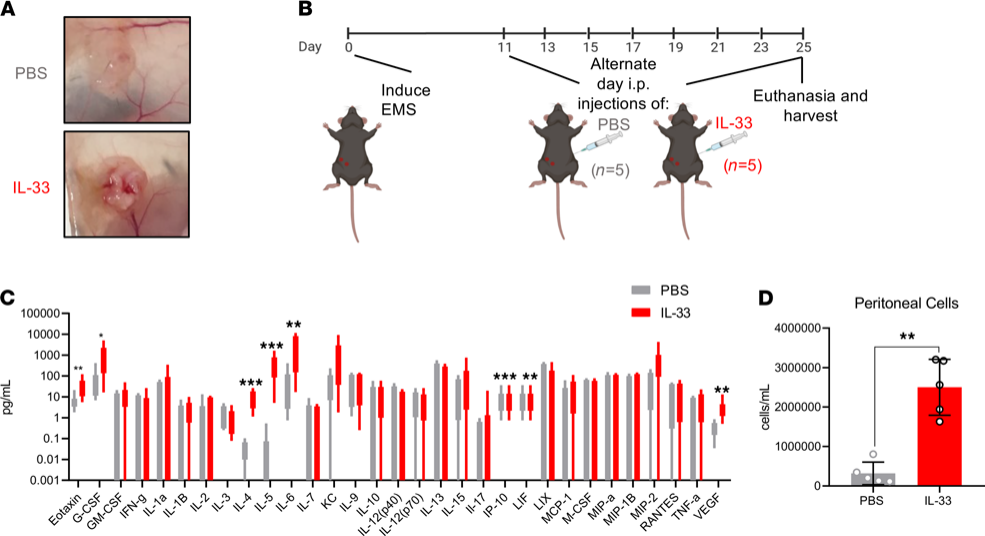
IL-33 induces inflammation and alters endometriosis lesion microenvironment in a murine model. (**A**) Representative images show exogenous IL-33 (*n* = 5) aggravates the lesion and contributes toward a larger, highly vascularized lesion compared with PBS-treated controls (*n* = 5). (**B**) Schematic representation of the experimental outline shows the induction of endometriosis (day 0), i.p. injections of either exogenous IL-33 or PBS on alternative days (beginning on day 11), and euthanasia (day 25). (**C**) Cytokines and chemokines from the peritoneal lavage fluid of PBS-treated and IL-33–treated mice were analyzed using a multiplex cytokine analysis. (**D**) Peritoneal lavage cells were harvested from PBS- and IL-33–treated EMS mice and counted using a Countess II FL Automated Cell Counter. **P < 0.01, ***P < 0.001. Mean ± SD. Nonparametric Student’s *t* test with Mann-Whitney.

While some patients with endometriosis remain asymptomatic, severe cases typically present with pathology and symptomology, including rich lesion innervation, debilitating chronic pelvic pain, and extensive pelvic adhesions (30–32). Here, we validated previous findings showing that the lesions of IL-33–treated mice appear larger and more vascularized compared with those of PBS-treated control mice (17) (Figure 2A; a detailed experimental outline is shown in Figure 2B).

While we previously demonstrated vast systemic inflammation in response to exogenous IL-33 (17), here we show that IL-33 drastically increased inflammation within the peritoneal cavity and lesion microenvironment. IL-33 stimulated the release of type 2 cytokines (IL-4 and IL-5), chemokines (CCL11 and CXCL10), macrophage-modulating cytokines (IL-6, LIF, G-CSF), and angiogenic cytokines (VEGF) (Figure 2C) in the PF. Exogenous IL-33 also drastically increased immune cell recruitment to the peritoneal and lesion microenvironment. WT C57BL/6 mice treated with IL-33 (*n* = 5) had significantly higher number of cells per mL in the PF compared with PBS-treated controls (*n* = 5) (Figure 2D).

**Figure 3:**
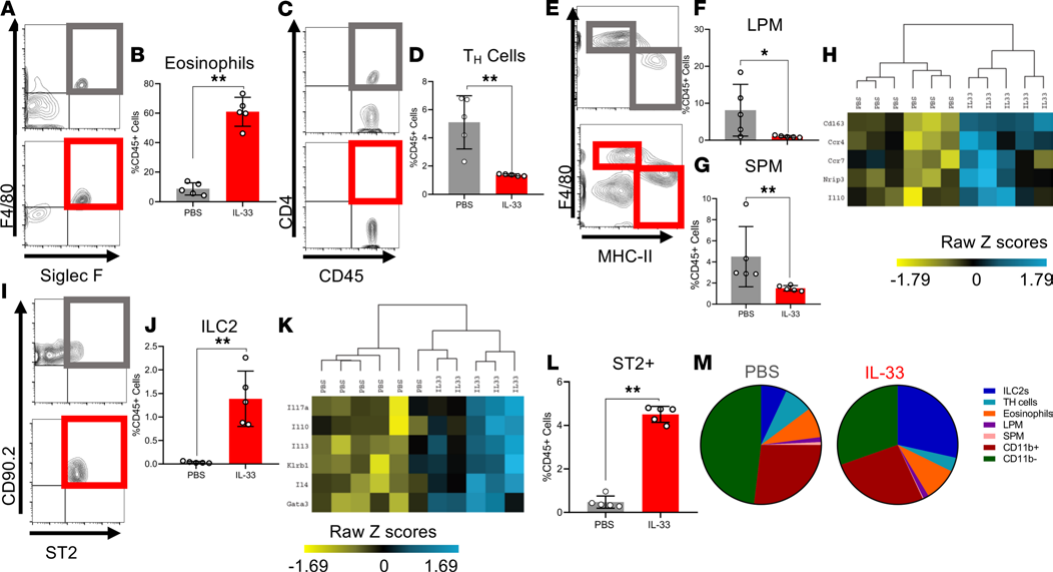
IL-33 alters the localized, peritoneal immune profile in EMS murine model. (**A**, **C**, **E**, and **I**) Representative flow cytometric images and gating strategy for immune profiling conducted on PF samples, including eosinophils, Th cells, LPM, SPM, and ILC2s in PBS-treated (*n* = 5) and IL-33–treated (*n* = 5) WT mice. (**A** and **B**) Frequency of eosinophils (singlet, live, CD45^+^CD11b^+^F4/80^+^, Siglec-F^+^). (**C** and **D**) Frequency of Th cells (singlet, live, CD45^+^CD11b^–^, F4/80^–^, CD4^+^). (**E** and **F**) Frequency of LPM (singlet, live, CD45^+^CD11b^+^Siglec-F^–^F4/80^hi^, MHC-II^lo^). (**E** and **G**) Frequency of SPM (singlet, live, CD45^+^CD11b^+^Siglec-F^–^F4/80^lo^, MHC-II^hi^). (**H**) Unsupervised hierarchical clustering using Euclidean distance and complete linkage of M2 alternative activation–associated genes expressed in RNA isolated from lesions of PBS- and IL-33–treated mice (Nanostring nSolver). (**I** and **J**) Frequency of ILC2 (singlet, live, CD45^+^ lineage^–^CD25^+^Thy2^+^, ST2^+^). (**K**) Unsupervised hierarchical clustering using Euclidean distance and complete linkage of ILC2-associated genes expressed in total RNA isolated from lesions of PBS- and IL-33–treated mice. (**L**) Proportion of ST2^+^ cells in the PBS- and IL-33–treated mice. (**M**) Pie graphs depicting the range of PF cells that are ST2^+^ in the PBS- and IL-33–treated mice. *P< 0.05, **P < 0.01. Mean ± SD. Nonparametric Student’s *t* test with Mann-Whitney.

In the PF obtained from IL-33–treated mice compared with PBS-treated controls, live CD45^+^F4/80^+^Siglec-F^+^ eosinophils were among the most significantly recruited immune cell (Figure 3, A and B). Furthermore, live CD45^+^CD11b^–^F4/80^–^CD4^+^ Th cells displayed a significantly lower proportion of CD45^+^ cells compared with PBS-treated controls (Figure 3, C and D). Interestingly, live CD45^+^CD11b^+^F4/80^int^MHCII^+^, small peritoneal macrophages (SPMs), which are bone marrow derived and differentiate from recruited peripheral monocytes (33), as well as live CD45^+^CD11b^+^F4/80^+^MHCII^–^, large peritoneal macrophages (LPMs), which are yolk sac derived and tissue resident macrophage (33), were significantly decreased in proportion (Figure 3, E–G). However, the expression of M2 macrophage genes evaluated by Nanostring transcriptomic profiling (including *CD163*, *Ccr4*, *Ccr7*, *Nrip3*, *IL10*) was significantly elevated in IL-33–treated lesions. Upon unsupervised hierarchical analysis, the IL-33 transcript profile clustered together and separated from the PBS lesion profile (Figure 3H). Live CD45^+^Lin^–^CD25^+^CD90.2^+^ST2^+^ ILC2s also composed a significantly larger proportion of the CD45^+^ cells within the peritoneal cavity compared with PBS-treated mice (Figure 3, I and J). The expression of ILC2 genes (including *IL17a, IL10, IL13, IL4, Klrb1, Gata3*) was elevated in the IL-33–treated lesions, and upon unsupervised hierarchical analysis, the IL-33 transcript profile clustered together and separated from the PBS lesion profile (Figure 3K).

Most relevant to the biology of ILC2, IL-33–treated mice also displayed a significant increase in the number of ST2^+^ immune cells in the peritoneal cavity, which suggests a positive feedback loop (Figure 3L). We sought to understand which cells were ST2^+^ in the PF of IL-33– and PBS-treated mice and therefore determine which ST2^+^ immune cells were increasing in abundance upon IL-33 stimulation. Accordingly, ST2^+^ ILC2s were the only significantly elevated ST2^+^ immune cell population (Supplemental Figure 4G). ST2^+^ Th cells, eosinophils, LPMs, and CD11b^+^ cells showed no change between PBS and IL-33 groups (Supplemental Figure 4, A–C, and E), and ST2^+^ SPMs and CD11b^–^ cells were significantly decreased in the PF (Supplemental Figure 4, D–F). Collectively, these data revealed that ILC2s were the only IL-33–responsive (ST2^+^) immune cell type that increased in abundance in response to exogenous IL-33 (overview shown Figure 3M).

### IL-33 alters murine endometriotic lesion architecture.

**Figure 4:**
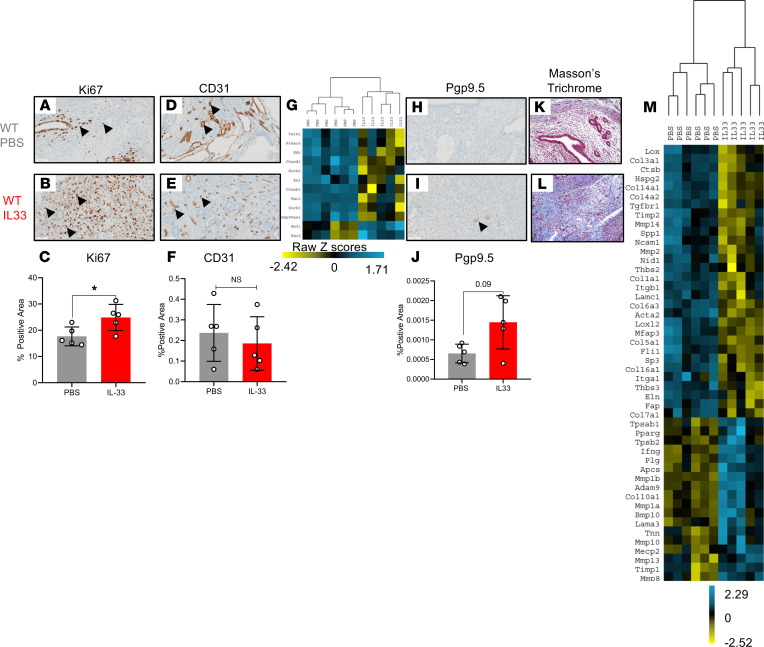
IL-33 alters EMS lesion proliferation, angiogenesis, innervation, and fibrosis. (**A** and **B**) Representative images of immunohistochemical staining using Ki67, a marker for proliferation, in PBS-treated (*n* = 5) and IL-33–treated (*n* = 5) mouse lesions. (**C**) Quantitative analysis of the percentage of positive area of Ki67^+^ cells. (**D** and **E**) Representative images of immunohistochemical staining using CD31, the marker for angiogenesis, in PBS- and IL-33–treated mouse lesions, respectively. (**F**) Analysis of the CD31^+^ area percentage in lesions from PBS- and IL-33–treated mice. (**G**) Unsupervised hierarchical clustering using Euclidean distance and complete linkage of angiogenesis-associated genes in RNA isolated from lesions of PBS- and IL-33–treated mice. (**H** and **I**) Representative images of immunohistochemical staining using the marker for innervation, Pgp9.5, in PBS- and IL-33–treated mouse lesions. (**J**) Analysis of the percentage of Pgp9.5^+^ area. The *P* value (*P* = 0.09) for this comparison is shown. (**K** and **L**) Representative images of Masson’s trichrome staining for collagen deposition in mouse lesions from PBS- and IL-33–treated groups. (**M**) Unsupervised hierarchical clustering using Euclidean distance and complete linkage of fibrosis-associated genes in RNA isolated from lesions of PBS- and IL-33–treated mice. Original magnification, ×200. Scale bar: 50 μm. *P < 0.05. Mean ± SD. Nonparametric Student’s *t* test with Mann-Whitney.

In addition to identifying the immune profile of the lesion microenvironment, we sought to understand how the endometriotic lesion itself changes in response to i.p. IL-33 stimulation. Our research group and others have previously demonstrated that IL-33 stimulates proliferation of the endometriotic lesion, a finding that we validate here (Figure 4, A–C). While IL-33 stimulated angiogenesis in vitro (17, 34), the lesions of IL-33–treated mice showed no difference in CD31 staining compared with PBS-treated mice (Figure 4, D–F). However, certain angiogenesis-associated genes showed a significant increase expression (including *Ncfl* and *Rac2*); however, other angiogenesis-associated genes showed a decrease in expression (including *Calm1*, *Prkaca*, *Kdr*, *Ctnnd1*, *Rock2*, *Axl*, *Ctnnb1*, *Rac1*, *Dock*, and *HSP90aa1*). The IL-33 angiogenesis transcript profile clustered together and separated from PBS-treated mice upon unsupervised hierarchical analysis (Figure 4G). This suggests that IL-33 has an impact on angiogenic pathways. Additionally, Pgp9.5, a neuronal marker, was substantially elevated in the lesions from IL-33–treated mice compared with PBS-treated controls (Figure 4, H–J). Finally, lesions from IL-33–treated mice had elevated collage deposition, depicted by the Masson’s trichrome staining (Figure 4, K and L). The lesions of IL-33–treated mice also had significant alterations to fibrosis-associated genes (Supplemental Table 1). Unsupervised hierarchical analysis revealed that the IL-33 transcript profile of fibrosis-associated genes clustered together and separated from the PBS transcript profile (Figure 4M). Therefore, exogenous i.p. IL-33 stimulates lesion proliferation, neurogenesis, and collagen deposition.

### IL-33–driven inflammation is ILC2 dependent.

Because we observed elevated ILC2s in the PF of patients with endometriosis and elevated ILC2s in IL-33–treated mice, and because the increase in ST2^+^ cells was solely due to the increase in ILC2s, we sought to determine if the inflammation, lesion proliferation, neuroangiogenesis, and fibrosis were dependent on ILC2s or if ILC2s were simply a bystander.

To do this, we used ILC2-deficient and -depleted mice. ILC-knockout mouse models are controversial in the field. The crude, yet highly effective, *RAG2^–/–^IL-2r*γ^*–/–*^ mice are ILC2 deficient and widely utilized. αCD90.2 is also used deplete ILC2s in *RAG2–/–* mice. However, the antibody depletion and corresponding ILC2 biology and ontogeny are not well studied; therefore, antibody depletion in a 25-day model, such as ours, can result in significant depletion but fully not deficient ILC2 populations. *RAG2–/–* mice, which contain functional ILC2s but lack T and B cells, were used as a control.

**Figure 5:**
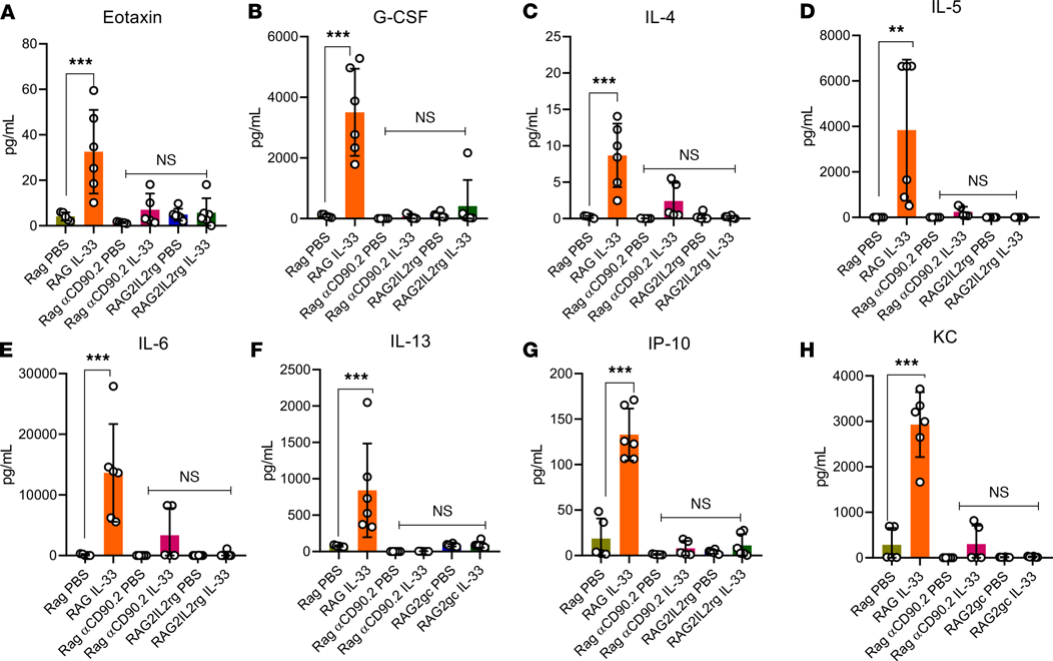
IL-33–driven inflammation is ILC2 dependent. *RAG2–/–* mice (ILC2 intact), *RAG2–/–*αCD90.2 mice (ILC2 depleted), and *RAG2^–/–^IL-2rγ–/–* mice (ILC2 deficient) were induced with endometriosis and underwent alternate day i.p. injections of PBS (*n* = 6) or IL-33 (*n* = 6). (**A**) IL-33–treated *RAG2–/–* mice had significantly elevated levels of eotaxin compared with PBS-treated *RAG2–/–*, PBS- and IL-33–treated *RAG2–/–*αCD90.2, and PBS- and IL-33–treated *RAG2–/–*IL-2rγ^–/–^ mice. (**B**) IL-33–treated *RAG2–/–* mice had significantly elevated levels of G-CSF compared with PBS-treated *RAG2^–/–^,* PBS- and IL-33–treated *RAG2–/–*αCD90.2, and PBS- and IL-33–treated *RAG2^–/–^IL-2rγ–/–* mice. (**C**) IL-33–treated *RAG2–/–* mice had significantly elevated levels of IL-4 compared with PBS-treated *RAG2^–/–^,* PBS- and IL-33–treated *RAG2–/–*αCD90.2, and PBS- and IL-33–treated *RAG2^–/–^IL-2rγ–/–* mice. (**D**) IL-33–treated *RAG2–/–* mice had significantly elevated levels of IL-5 compared with PBS-treated *RAG2^–/–^,* PBS- and IL-33–treated *RAG2–/–*αCD90.2, and PBS- and IL-33–treated *RAG2^–/–^IL-2rγ–/–* mice. (**E**) IL-33–treated *RAG2–*^/–^ mice had significantly elevated levels of IL-6 compared with PBS-treated *RAG2^–/–^,* PBS- and IL-33–treated *RAG2–/–*αCD90.2, and PBS- and IL-33–treated *RAG2^–/–^IL-2rγ–/–* mice. (**F**) IL-33–treated *RAG2–*^/–^ mice had significantly elevated levels of IL-13 compared with PBS-treated *RAG2^–/–^,* PBS- and IL-33–treated *RAG2–/–*αCD90.2, and PBS- and IL-33–treated *RAG2^–/–^IL-2rγ–/–* mice. (**G**) IL-33–treated *RAG2–/–* mice had significantly elevated levels of IP-10 compared with PBS-treated *RAG2^–/–^,* PBS- and IL-33–treated RAG2^–/–^αCD90.2, and PBS- and IL-33–treated RAG2^–/–^IL-2rγ^–/–^ mice. (**H**) IL-33–treated *RAG2–/–* mice had significantly elevated levels of CXCL1/KC compared with PBS-treated *RAG2^–/–^,* PBS- and IL-33–treated *RAG2–/–*αCD90.2, and PBS- and IL-33–treated *RAG2^–/–^IL-2rγ–/–* mice. **P < 0.01, *P* < 0.0001. Mean ± SD. One-way ANOVA was used.

After inducing endometriosis, the *RAG2–/–* mice (ILC2 intact), *RAG2–*^/–^αCD90.2 mice (ILC2 depleted), and *RAG2^–/–^IL-2r*γ^*–/–*^ mice (ILC2 deficient) were treated with PBS and IL-33. The *RAG2–/–* mice (ILC2 intact) exhibited a similar inflammatory profile to the WT C57BL/6 mice treated with IL-33: drastic increases in inflammation, including type 2 cytokines (IL-4, IL-5, and IL-13), chemokines (CXCL10, CXCL1, and CCL11), and immune-modulating cytokines (IL-6, GM-CSF, and G-CSF). Interestingly, both the *RAG2–/–*αCD90.2 mice (ILC2 depleted) and *RAG2^–/–^IL-2r*γ^*–/–*^ mice (ILC2 deficient) treated with IL-33 had no differences in eotaxin (Figure 5A), G-CSF (Figure 5B), IL-4 (Figure 5C), IL-5 (Figure 5D), IL-6 (Figure 5E), IL-13 (Figure 5F), IP-10 (Figure 5G), or CXCL1/KC (Figure 5H). This suggests that IL-33–driven inflammation requires intact ILC2s.

### IL-33–driven immune cell recruitment and alterations is ILC2 dependent.

**Figure 6:**
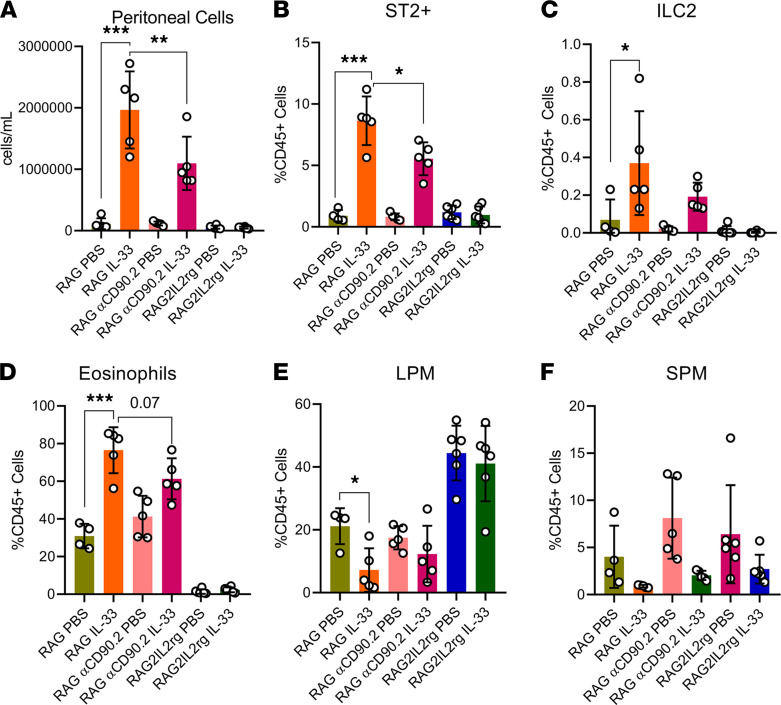
IL-33–driven immune cell dysfunction is ILC2 dependent. *RAG2–*^/–^ mice (ILC2 intact), *RAG2–/–*αCD90.2 mice (ILC2 depleted), and *RAG2^–/–^IL-2rγ–/–* mice (ILC2 deficient) were induced with endometriosis and underwent alternate day i.p. injections of PBS (*n* = 6) or IL-33 (*n* = 6). (**A**) Peritoneal lavage cells harvested from PBS- and IL-33–treated EMS mice were counted using a Countess II FL Automated Cell Counter. (**B**) Frequency of ST2^+^ cells in the PBS- and IL-33–treated mice analyzed via flow cytometry. (**C**) Frequency of ILC2 (singlet, live, CD45^+^Lineage^–^CD25^+^Thy2^+^, ST2^+^). (**D**) Frequency of eosinophils (singlet, live, CD45^+^CD11b^+^F4/80^+^, Siglec-F^+^). (**E**) Frequency of LPM (singlet, live, CD45^+^ CD11b^+^ Siglec-F^–^ F4/80^hi^, MHC-II^lo^). (**F**) Frequency of SPM (singlet, live, CD45^+^CD11b^+^ Siglec-F^–^F4/80^lo^, MHC-II^hi^). *P < 0.05, **P < 0.01, ***P < 0.001. Mean ± SD. One-way ANOVA was used.

Similarly, the *RAG2–/–* mice (ILC2 intact) treated with IL-33 showed drastic increases in peritoneal cell numbers (Figure 6A) and ST2^+^ cells (Figure 6B), as well as a prominent increase in ILC2s (Figure 6C), a stark increase in eosinophils (Figure 6D), a decrease in LPMs (Figure 6E), and a decrease in SPMs (Figure 6F). However, in the *RAG2–/–*αCD90.2 mice (ILC2 depleted) treated with IL-33, we saw a significant decrease in peritoneal cell numbers (Figure 6A) and ST2^+^ cells (Figure 6B), as well as decreased ILC2s (Figure 6C) compared with the IL-33–treated *RAG2–/–* mice (ILC2 intact). That corresponded with less eosinophil recruitment (Figure 6D) and more LPMs and SPMs (Figure 6, E and F), compared with the IL-33–treated *RAG2–/–* (ILC2 intact). Using the *RAG2^–/–^IL-2r*γ^*–/–*^ mice (ILC2 deficient), we observed no difference between PBS and IL-33 in peritoneal cell numbers (Figure 6A) and ST2^+^ cells (Figure 6B). Additionally, no ILC2s were found (Figure 6C), no IL-33–induced influx of eosinophils was observed (Figure 6D), and there were no IL-33–driven changes in LPM or SPM populations (Figure 6, E and F). This demonstrates that intact ILC2s are required for the IL-33–driven immune cell influx and the specific IL-33 immune alterations.

### IL-33–driven lesion proliferation and fibrosis is ILC2 dependent.

**Figure 7:**
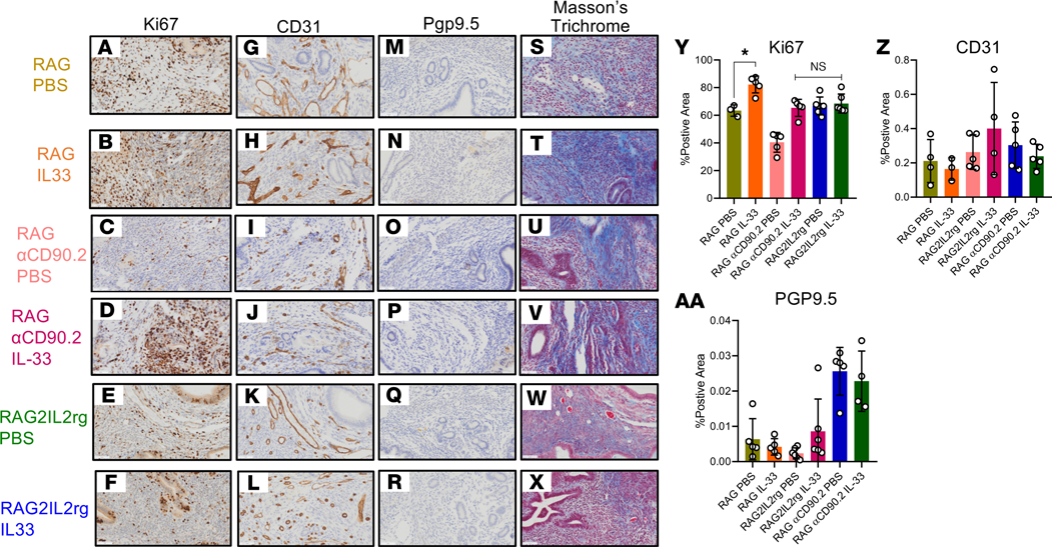
IL-33–driven lesion proliferation and fibrosis is ILC2 dependent. *RAG2–/–* mice (ILC2 intact), *RAG2–/–*αCD90.2 mice (ILC2 depleted), and *RAG2^–/–^IL-2rγ–/–* mice (ILC2 deficient) were induced with endometriosis and underwent alternate day i.p. injections of PBS (*n* = 3-6) or IL-33 (*n* = 4-6). (**A–F**) Representative immunohistochemical staining and quantitative analysis of lesion Ki67 staining. (**G–L**). Representative immunohistochemical staining and quantitative analysis of lesion CD31 staining. (**M–R**). Representative immunohistochemical staining and analysis of lesion Pgp9.5 staining. (**S–X**) Representative immunohistochemical staining and analysis of lesion Masson’s trichrome staining. Original magnification, ×200. Scale bar: 50 μm. (**Y**) Analysis of the percentage of Ki67^+^ area. (**Z**) Analysis of the percentage of CD31^+^ area. (**AA**) Analysis of the percentage of Pgp9.5^+^ area. *P < 0.05. Mean ± SD. One-way ANOVA was used.

Additionally, the *RAG2–/–* mice (ILC2 intact) treated with IL-33 showed drastic increases in Ki67 staining (Figure 7, A, B, and Y) and increases in collagen deposition (Figure 7, S and T) compared with the PBS-treated mice, which is similar to that seen in WT mice. Interestingly, both the *RAG2–/–*αCD90.2 mice (ILC2 depleted) and *RAG2^–/–^IL-2r*γ^*–/–*^ mice (ILC2 deficient) exhibited no IL-33–driven Ki67 staining (Figure 7, C–F and Y) or collagen deposition (Figure 7, U–X). This shows that both the IL-33–driven lesion proliferation and collagen deposition (by extension fibrosis) are ILC2 mediated.

However, there seemed to be no IL-33–driven angiogenic effect (as seen in the lack of changes in CD31 staining) (Figure 7, G–L and Z), and the IL-33–driven neurogenesis effect (by Pgp9.5 staining) may not be mediated by ILC2s but perhaps other molecular pathways (Figure 7, M–R and AA).

### Targeting IL-33 through neutralizing antibody is a viable therapeutic target.

**Figure 8:**
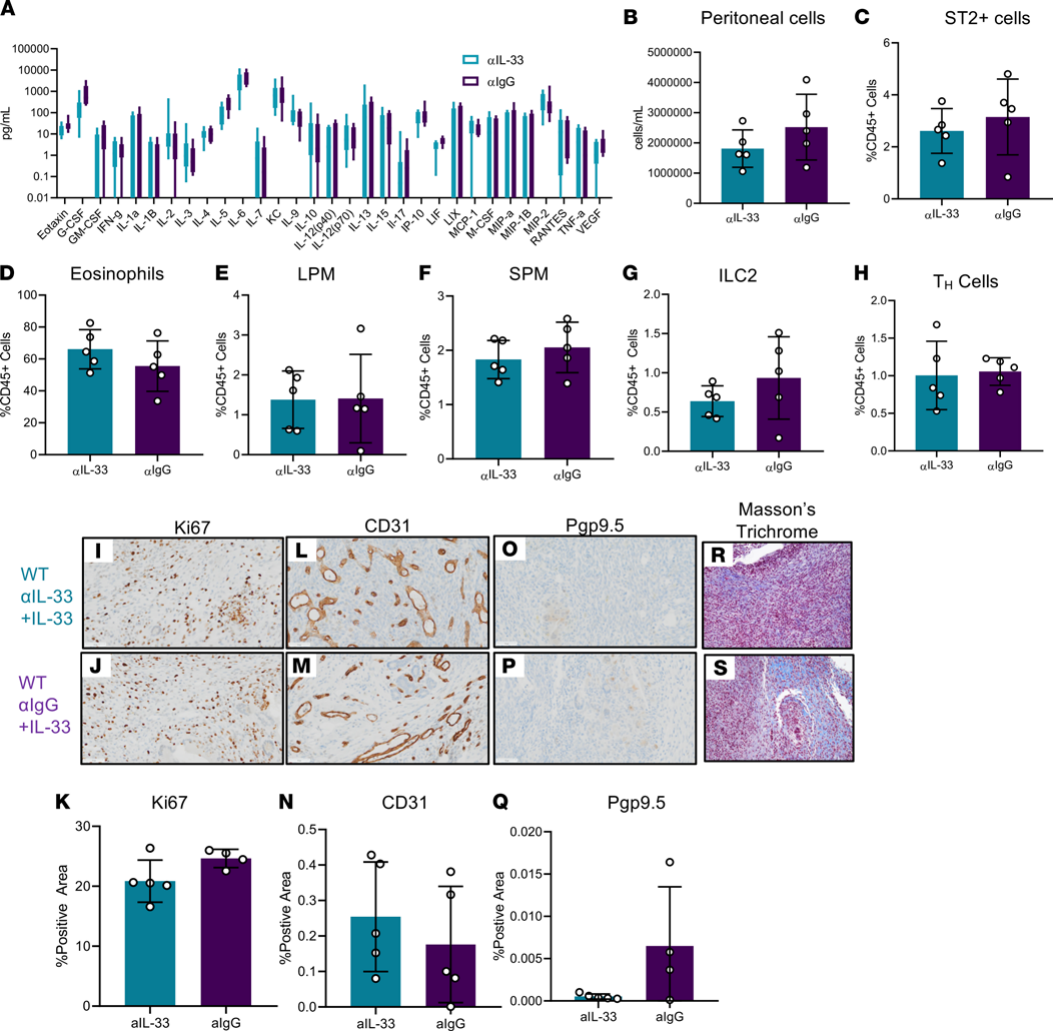
IL-33 Neutralization represents a promising therapeutic avenue in EMS. WT mice were induced with endometriosis and underwent alternate day i.p. injections of IL-33 along with either a neutralizing IL-33 antibody (αIL-33) (*n* = 5) or IL-33 and IgG control antibody (αIgG) (*n* = 5). (**A**) Cytokine and chemokine analysis in PF samples from αIL-33– or αIgG-treated EMS mice. (**B**) Peritoneal lavage cells harvested from αIL-33– or αIgG-treated EMS mice counted using a Countess II FL Automated Cell Counter. (**C**) Frequency of ST2^+^ cells in the αIL-33– or αIgG-treated WT mice analyzed via flow cytometry. (**D**) Frequency of eosinophils (singlet, live, CD45^+^CD11b^+^F4/80^+^, Siglec-F^+^). (**E**) Frequency of LPM (singlet, live, CD45^+^CD11b^+^Siglec-F^–^F4/80^hi^, MHC-II^lo^). (**F**) Frequency of SPM (singlet, live, CD45^+^CD11b^+^Siglec-F^–^F4/80^lo^, MHC-II^hi^). (**G**) Frequency of ILC2 (singlet, live, CD45^+^lineage^–^CD25^+^Thy2^+^, ST2^+^). (**H**) Frequency of Th cells. (**I–K**) Representative immunohistochemical staining and quantitative analysis of lesion Ki67 staining. (**L–N**) Representative immunohistochemical staining and quantitative analysis of lesion CD31 staining. (**O**–**Q**) Representative immunohistochemical staining and analysis of lesion Pgp9.5 staining. (**R** and **S**) Representative histochemical staining of lesion Masson’s trichrome staining. Original magnification, ×200. Scale bar: 50 μm. Mean ± SD. Nonparametric Student’s *t* test with Mann-Whitney.

Thus far, we observed a consistent pathological effect of IL-33 in our murine model of endometriosis, and this pathological effect is dependent on ILC2s. Therefore, IL-33 seems to be a promising, nonhormonal therapeutic target for treating endometriosis. As a proof of concept, we induced endometriosis in WT mice and treated them with IL-33 in addition to either a neutralizing antibody or isotype control. We found that adding a neutralizing antibody following IL-33 treatment (*n* = 5) slightly but not significantly reduced peritoneal inflammation (Figure 8A), reduced peritoneal cell concentration (Figure 8B), and reduced relative abundance of ST2^+^ cells (Figure 8C) compared with isotype control (*n* = 5). The abundance of eosinophils (Figure 8D), LPMs (Figure 8E), SPMs (Figure 8F), ILC2s (Figure 8G), and Th cells (Figure 8H) did not change significantly in response to neutralization. Additionally, we observed no changes in Ki67 staining (Figure 8, I–K) or CD31 staining (Figure 8, L–N). However, both Pgp9.5 staining (Figure 8, O–Q) and Masson’s trichrome staining (Figure 8, R and S) exhibited decreased staining in the αIL-33–treated mice compared with αIgG controls.

## Discussion

Despite decades of research, endometriosis remains an enigmatic disorder with chronic inflammation as a common denominator contributing to the disease pathophysiology and its associated symptomology. Consequently, current treatments mainly use nonspecific approaches (surgery, menstruation suppression, and symptom management) without addressing the underlying pathological mechanisms that drive the disease. The current treatment standards demonstrate a desperate need for the study of complex immune mechanisms in the context of endometriosis. Data presented here demonstrate for the first time to our knowledge the expansion of a unique ST2^+^ ILC2 population in the PF of patients with endometriosis. Data presented here also demonstrate that IL-33 colocalizes with both epithelial and stromal cells within the endometriotic lesion. This colocalization pattern indicates that IL-33 likely does not form stromal or epithelial niches with ILC2s (as is the case of lung or adipose tissue; refs. 20, 35), but both epithelium- and stroma-associated IL-33 were found throughout the endometriotic lesion. We cannot completely exclude the possibility that such IL-33–ILC2s niches exist in the endometriotic lesion, as further studies using different types of lesions, such as endometriomas, deep infiltrating endometriosis, and superficial peritoneal lesions, must be carried out to shed light on the IL-33 and ILC2 biology within lesions.

Using our well-established syngeneic murine model of endometriosis, we unravel how IL-33 and ILC2s contribute to the pathology of endometriosis. Previously, we showed that IL-33 induced systemic inflammation and lesion proliferation (17), while others demonstrated that ST2-deficient mice had significantly smaller lesions and less lesion proliferation (26). Here, we show that IL-33 significantly perpetuated local peritoneal inflammation, which is a key hallmark of endometriosis. The vast recruitment of immune cells to the peritoneal cavity and significant increase in type 2 cytokines (including IL-4 and IL-5), immune-modulating cytokines (such as IL-6 and G-CSF), and angiogenic factors (such as VEGF) suggest that IL-33 actively contributes to a pathological inflammatory microenvironment similar to that observed in patients with endometriosis. Importantly, IL-33 treatment increased the total number of ST2^+^ immune cells. However, of the ST2^+^ cells, only ILC2s were found to be significantly increased. Data presented here also demonstrate that IL-33 stimulates profound alterations to the lesion architecture, including increases in proliferation, neurogenesis, and fibrosis. Highly fibrotic lesions and intralesion neuronal markers have been correlated with pain severity and advanced disease (36). Therefore, IL-33 perpetuates multiple key hallmarks of disease severity, including inflammation, immune cell recruitment, proliferation, neurogenesis, and fibrosis. We highlight, however, that determining the precise role of IL-33 in the healthy endometrium, menstruation, implantation, and partition warrants further investigation.

The remaining focus of our investigation was to determine whether the IL-33–driven inflammation and lesion alterations were in fact ILC2 dependent. Indeed, we provide concrete evidence that the IL-33–driven severe pathology observed in the C57BL/6 WT and *RAG2–/–* mice (ILC2 intact) was significantly reduced in the IL-33–treated *RAG2–/–*αCD90.2 mice (ILC2 depleted) and completely absent in the IL-33–treated *RAG2^–/–^IL-2r*γ^*–/–*^ mice (ILC2 deficient). Therefore, using two separate methodologies, we show that IL-33–induced inflammation, immune cell recruitment, lesion proliferation, and fibrosis are indeed ILC2 dependent in the endometriosis murine model. However, the IL-33–driven neurogenesis of the lesion is likely ILC2 independent.

Because ILC2s are tissue-resident immune cells and do not migrate significantly after the postnatal period (37), we propose that peritoneal IL-33 does not recruit ILC2s to the lesion but rather stimulates tissue-resident ILC2s to proliferate, activate, and produce large quantities of cytokines, including eotaxin, IL-4, and IL-5. We pinpoint ILC2s as the source of these 3 peritoneal cytokines, because lineage tracing of ILC2s residing in lung and adipose tissue have shown that ILC2s produce IL-4, IL-5, and eotaxin (38). Further, when we treated both *RAG2–/–*αCD90.2 mice (ILC2 depleted) and *RAG2^–/–^IL-2r*γ^*–/–*^ mice (ILC2 deficient) with IL-33, we did not find significantly elevated levels of eotaxin, IL-4, and IL-5. Given that the transcriptomic analysis of endometriotic lesions from IL-33–treated WT mice revealed significantly elevated expression of ILC2-associated genes, including *IL4*, *IL13*, *GATA3*, *Klrb1*, *Il10*, and *Il17a*, and considering human endometriotic lesions are ST2^+^ (17), we propose that ILC2s likely reside within the lesion itself. IL-33–responsive ILC2s reside in both the mesothelium of the peritoneal cavity (38) as well as the uterus (39); therefore, either the mesothelium or the uterus could be the source of ILC2s in the endometriotic lesions. However, further investigation is required to understand the precise origin of ILC2s that may reside within endometriotic lesions.

Many studies have implicated the essential role of macrophages in the establishment and progression of endometriosis as they facilitate adhesion, neuroangiogenesis, and fibrosis: specifically SPMs and M2 macrophages (40–44). Recent in vitro studies revealed that IL-33 derived from endometriotic stromal cells polarizes macrophages to an M2 phenotype, which the authors conclude worsens disease (18, 45). However, in adipose and lung tissue, it has been repeatedly shown that IL-33 activates ILC2s to produce type 2 cytokines, including IL-4, IL-5, and IL-13, which polarize macrophages to an M2 phenotype (28, 46). Here, we demonstrated that the relative abundance of both LPMs and SPMs were decreased in IL-33–treated WT and *RAG2–/–* mice (ILC2 intact). However, despite being decreased in abundance, transcriptional analysis of the lesion revealed that SPM differentiation genes, including *IRF4*, and M2 polarizing–associated genes, including *CD163*, *Ccr4*, *Ccr7*, *Nrip3*, and *IL10*, were elevated in IL-33–treated WT and *RAG2–/–* mice (ILC2 intact). Therefore, we suggest that the macrophages are likely M2 polarized in response to IL-33 and are responsible for contributing to fibrosis and tissue remodeling through macrophage-associated fibrosis genes, including *Mmp13* and *Tnn*. Interestingly, the LPM and SPM populations were largely ST2^–^. This suggests that IL-33 is likely not acting directly on the macrophage to polarize them to an M2 phenotype but rather IL-33 acts on ILC2s to produce large quantities of IL-4, IL-5, and IL-17, which in turn polarize the macrophages to an M2 phenotype (44). Additionally, the high levels of IL-5 and eotaxin produced by ILC2s likely contributed to eosinophil recruitment to the peritoneal cavity. While eosinophils have not been extensively studied in the context of endometriosis, eotaxin and IL-5 are elevated in the PF of patients with severe endometriosis (9). Furthermore, alarmin activated eosinophils in collaboration with ILC2s and IL-4, IL-13 activated macrophages drive fibrosis and inflammatory fibrotic diseases (47). Taken together, our data suggests that IL-33–activated ILC2s produce cytokines, including IL-4, IL-5, and eotaxin, which recruit eosinophils and activate macrophages and then drive fibrosis and other alterations to the architecture of the lesions.

This makes the IL-33/ST2 axis one of the most promising nonhormonal therapeutic targets for endometriosis. In fact, recruitment of patients with endometriosis is currently ongoing for a phase II clinical trial to assess the efficacy of a monoclonal antibody targeting IL-33 (MT-2990, Mitsubishi Tanabe Pharma Corporation; https://clinicaltrials.gov/ct2/show/NCT03156738). To gain further mechanistic insight, we neutralized IL-33 in our syngeneic mouse model of endometriosis using AF3626, a mouse IL-33–neutralizing antibody. Our results did not reveal major differences in inflammatory or lesion architecture between the IL-33–neutralizing antibody–treated and control mice, in contrast to reports in the recent study by Kato et al. (26). However, this could be due to the dose and/or efficacy of the neutralizing antibody, which could be optimized. One of the major differences between Kato et al.’s published study and ours is that Kato et al. used human recombinant IL-33 and human IL-33–neutralizing antibody and tested their efficacy in a BALB/c mouse model of endometriosis, whereas we used mouse recombinant IL-33 and mouse IL-33–neutralizing antibody in our mouse model. However, we still believe the IL-33/ST2 axis is a promising therapeutic target, given the multitude of processes and immune functions governed by this axis.

In conclusion, we demonstrate the presence of ST2^+^ ILC2s in the lesion microenvironment of patients with endometriosis and that IL-33–driven ILC2 expansion plays a vital role in the pathophysiology of endometriosis. Our data reveal that effects of IL-33 are most likely governed through ILC2s and modulation of Th2 type cytokine response, but we cannot exclude the possibility that IL-33 may directly act on other cell types that express ST2. While our studies were the first to our knowledge to reveal the molecular basis of how IL-33 in concert with ILC2s potentially drives endometriosis pathology, we acknowledge some of our study’s limitations, which are likely inherent to the field of endometriosis in general. It is not possible to recapitulate all the classic features of human endometriosis, such as hormonal dependence, chronic inflammation, pain, and fibrosis, as well as types and stages of the disease in a mouse model. We also acknowledge the lively debate in the literature on a suitable mouse model to study ILC2 biology and lineage tracing. Our study, however, objectively provided evidence that IL-33 and ILC2s, including those not activated by IL-33, are of critical importance in the pathophysiology of endometriosis, which should be studied further, and that this axis is a promising, nonhormonal therapeutic target for the field.

## Methods

### Patients.

Patients with endometriosis who were undergoing surgical resection due to endometriosis-associated infertility at Greenville Hospital (Greenville, South Carolina, USA) and Kingston Health Sciences Centre (Kingston, Ontario, Canada) were recruited and included in the study. The disease stage was assessed by the attending physician during surgical resection, as per American Society for Reproductive Medicine criteria (48). Healthy, fertile controls undergoing elective tubal ligations at Kingston Health Sciences Centre were recruited. All patients enrolled in the study were free of hormonal therapy for at least 3 months prior to sample collection. PF was collected by washing the peritoneum with PBS, avoiding peripheral blood contamination. Both tissue samples and PF supernatant were stored at −80°C prior to processing.

### Flow cytometry on human PF.

Frozen PF cells were thawed in a 37°C water bath and quickly washed in PBS with 5% FBS. Cells were incubated with 100 μg/mL DNase 1 (Roche, 1014159001) at 4°C for 10 minutes, washed with PBS with 5% FBS, and passed through the 70 μm strainer. For surface marker staining, 10^6^ cells per sample were incubated with the following monoclonal antibodies purchased from Biolegend: Pacific blue–CD45 (HI30; 1:75), FITC-CD127 (A019D5; 1:30), PE-ST2 (polyclonal, R&D Systems), APC-CD3 (HIT3a), APC-CD14 (63D3), APC-CD11b (M1/70), APC-CD11c (3.9), APC-CD19 (HIB19), APC-TCRα/β(IP26), APC-TCRγ/δ (B1), APC/Cy7-CD294 (BM16), and Zombie Aqua Fixable Viability Kit (Biolegend, 423101). Stained cells were analyzed on a Cytoflex S (Beckman Coulter), and the data were analyzed with FlowJo software (TreeStar). Live gate was set with one-half heat-killed viability control. Gates were set using fluorescence minus ones (FMOs).

### Immunofluorescence on human endometriosis lesions.

Frozen endometriotic lesions were embedded in optimal cutting temperature compound and sectioned at a 5 μm thickness. Then, the sections were washed with PBS, permeabilized with 0.2% Triton X-100, washed with PBS for 10 minutes, and blocked with 10% FBS for 1 hour. The sections were then incubated with primary antibodies overnight at 4°C, washed with PBST, and then, where required, stained with Alexa Fluor–conjugated secondary antibodies for 1 hour at room temperature. The following primary antibodies were used: Goat Anti-human IL-33 (1:40, R&D Systems, AF3625), Mouse Anti-human Vimentin (1:400, Cell Signaling Technology, clone D21H3), Mouse Anti-Pan cytokeratin (1:40, Cell Signaling Technology, clone AE1/AE3), Rabbit Anti-goat Alexa Fluor 647 (1:200; Invitrogen, A27018). Immunostained tissue sections were imaged using the Leica TCS SP8 confocal microscope. Images were taken in a *Z*-stack (1.20 μm; *Z* dimension) and tile scan (12 tiles, 580 μm × 435 μm each tile; *x* and *y* dimensions) acquisition mode. Two to 3 fields per preparation were imaged using HC PL APO CS2 ×63/1.40 oil objective and LAS-X Software (Leica Microsystems).

### Mice.

WT C57BL/6 mice were purchased from Charles River, and *RAG2–/–* mice (B6.129S6-*Rag2tm1Fwa* N12; model RAGN12-F) and RAG2IL-2rγ–deficient mice [C57BL/6NTac;B10(Cg)-*Rag2^tm1Fwa^ Il2rgtm1Wjl*; model 4111-F] were purchased from Taconic Biosciences. Mice were maintained at room temperature in conventional light/dark cycles. WT mice were kept in conventional housing and RAG2-deficient and RAG2IL-2rγ–deficient mice were kept in sterile barrier housing. All studies were done with 8- to 10-week-old female mice.

### Murine model of endometriosis.

C57BL/6 WT, *RAG2–/–*, and *RAG2^–/–^IL-2r*γ^*–/–*^ mice underwent the same procedure to induce endometriosis (timeline outlined in Figure 2B, created with BioRender.com). Briefly, endometriosis was surgically induced by grafting 3 mm donor uterine explants into recipient mouse abdomens, as per our well-established protocol (44). The mice were rested for 11 days following surgery. Experimental mice were then treated with i.p. injection of exogenous recombinant mouse IL-33 at 1 μg/mouse (Biolegend, 580506) every other day for 14 days. Control mice received PBS. Mice were euthanized, peritoneal lavage was collected using ice-cold PBS, and lesions were photographed and then harvested. PF was centrifuged at 400*g* and stored at −80°C. PF samples underwent cytokine and chemokine analysis using a commercially available murine multiplex cytokine and chemokine array (Eve Technologies, MD31). PF cells were resuspended in a 1:10 dilution of DMSO to FBS, frozen at a controlled rate of –1°C per minute, and then stored in liquid nitrogen. Endometriotic lesions were stored in 4% paraformaldehyde for 16 hours, transferred to 70% ethanol, and then embedded in paraffin blocks or snap frozen in liquid nitrogen and stored at –80°C until RNA extraction.

### ILC2 antibody depletion.

In *RAG2–/–* mice, endometriosis was induced as described above. The mice were rested for 7 days and then underwent 3 consecutive days of 200 μg i.p. CD90.2 antibody (Bio X cell, 30H12) treatment to deplete ILC2s as per previously published methodology (49). Experimental mice were then treated with i.p. injection of exogenous recombinant mouse IL-33 at 1 μg per mouse (Biolegend, 580506) every other day for 14 days while the control mice received PBS. The same processing for tissues was conducted.

### IL-33 neutralization.

Endometriosis was induced in C57BL/6 mice as described above. All mice were treated with i.p. injections of exogenous recombinant mouse IL-33 at 1 μg per mouse (Biolegend, 580506) at the same dosing schedule as above; however, experimental mice were also in tandem treated i.p. with goat anti-mouse IL-33 antibody at 2.5 μg per mouse (R&D Systems, AF3626) and appropriate isotype control (R&D Systems, AB-1080C). The same processing for tissue harvest was conducted.

### Flow cytometry on murine PF.

Frozen PF cells were thawed in a 37°C water bath and immediately washed in PBS and 5% FBS. Cells were incubated with 100 μg/mL DNase 1 (Roche, 1014159001) at 4°C for 10 minutes. Cells were then passed through a 70 μm strainer to create a single-cell suspension. For surface marker staining, 5 × 10^5^ cells/sample were incubated with the following monoclonal antibodies purchased from Biolegend, unless otherwise stated: Pacific blue–CD11b (M170), Superbright600-ST2 (RMST2-2), Bright Violet 785-CD45 (30-F11), FITC-F4/80 (BM8), PE-Siglec-F (E50-2440), PE/Cy7-FCER1 (MAR-1), APC-CD117 (2B8), Alexa Fluor 700-CD4 (RM4-5), APC/Cy7-MHC-II (M5/114.15.2), Pacific blue–CD90.2 (53.2.1), Brilliant Violet 785- CD45 (30-F11), PE-ST2 (Thermo Fisher Scientific, RMST2-2), FITC-CD25 (PC61), APC-CD3ε (145-2C11), APC-CD4 (RM4-5), APC-CD19 (1D3), APC-NK1.1 (PK136), APC-FCER1 (MAR-1), APC-Gr-1 (RB6-8C5), APC-CD11b (M1/70), Alexa Fluor 700–CD127 (Thermo Fisher Scientific, A7R34), and Zombie Aqua Fixable Viability Kit (no. 423101). Stained cells were analyzed on a Cytoflex S (Beckman Coulter), and the data were analyzed with FlowJo software (TreeStar). Live gate was set with one-half heat-killed viability control. Gates were set using FMOs. Full gating strategies are presented in Supplemental Figure 3.

### Gene expression with Nanostring nCounter mouse fibrosis V2 panel and associated statistical analysis.

Total RNA was extracted from frozen WT mouse lesions treated with PBS (*n* = 6) or IL-33 (*n* = 5) using the Norgen biotek Total RNA isolation kit, as per the manufacturer’s instructions. Briefly, tissues were disrupted using Bead Ruptor 23 (Omni International) and 600 μL lysate buffer and then were centrifuged at 14,000*g* for 1 minute. The supernatant was mixed with equal parts 70% ethanol, passed through columns, and eluted. The concentration of RNA was quantified using a Nanodrop 2000 Spectrophotometer. All samples were normalized to 20 ng/μL and analyzed using Nanostring nCounter and nCounter Digital Analyzer by means of a nCounter Mouse Fibrosis V2 Panel, which consists of 760 genes and 10 housekeeping genes.

The resultant RCC files and provided RLF file were uploaded to Nanostring’s nSolver platform. The raw data were exported as a CSV file and uploaded into MATLAB (2019b). Unsupervised hierarchical clustering using Euclidean distance and the complete linkage method was used to check for outliers (Supplemental Figure 5). The raw data were normalized using the geomean of positive controls as well as housekeeping genes with robust gene expression and a percentage of coefficient of variation of ≤65.

### Histochemistry and immunohistochemistry of murine lesion.

Paraffin-embedded lesions were sectioned at 5 μm, and antigen retrieval was conducted. Sections were then stained with rabbit anti-mouse ki67 (Abcam, ab1558, 1:1000), rabbit anti-mouse CD31 (New England Biolabs, 77699S, 1:100), or rabbit anti-mouse Pgp9.5 (New England Biolabs, 13179S, 1:800) using the Department of Pathology’s Ventana Discovery Immunostainer (Ventana Medical Systems Inc.) at Queen’s University. Anti-rabbit secondary antibodies were then stained for 1 hour, and the Ultrablue DAB detection kit was used (Ventana Medical Systems Inc.). Masson’s trichrome slides were counterstained for 4 minutes with hematoxylin and bluing reagent, and a coverslip was applied. An Aperio ScanScope SC slide scanner (Leica Biosystems Imaging Inc.) was used to take images, and then images were quantified using Halo image analysis platform Indica Lab Inc.). Analysis of Ki67 was quantified using Halo’s CytoNuclear v1.6 algorithm, whereas CD31 and Pgp9.5 were quantified using Halo’s Area Quantification v1.0.

### Statistics.

Unpaired 2-tailed *t* test with Welch’s correction was used when 2 independent, nonparametric groups needed to be assessed for statistical significance. One-way ANOVA was used when multiple independent nonparametric groups needed to be assessed for statistical significance. GraphPad Prism 8.4 software was used. *P* values of less than 0.05 were considered significant.

### Study approval.

The Health Sciences Research Ethics Board at Queen’s University and Greenville Health System (Greenville, South Carolina, USA) approved all studies where human samples were used. Written informed consent was obtained from all patients prior to human sample collection and storage. All animal studies were approved by the Queen’s University Animal Care Committee.

## Author contributions

JEM and CT conceived and designed the experiments. JEM performed all experiments, analyzed the data, and wrote the manuscript. HL and LKM helped implement and performed the experiments. OB, SLY, and BAL conducted patient sample collection and processing. MK provided materials, protocols, and advice for the experiments. CT supervised the study and wrote the manuscript.

## Supplementary Material

Supplemental data

## Version 1 10/26/2021

In-Press Preview

## Version 2 12/08/2021

Electronic publication
