# Supplementary material for: IL-33 activates group 2 innate lymphoid cell expansion and modulates endometriosis
Source: JCI Insight. 2021 Dec 8;6(23):e149699. doi: 10.1172/jci.insight.149699 (PMC8675188; doi:10.1172/jci.insight.149699)
Supplement: Supplemental data [file jciinsight-6-149699-s199.pdf]

Supplementary Figures

Interleukin-33 activates group 2 innate lymphoid cell expansion and modulates endometriosis

Jessica E. Miller[1], Harshavardhan Lingegowda[1], Lindsey K. Symons[1], Olga Bougie[2], Steven L. Young[3], Bruce A. Lessey[4], Madhuri Koti[1,2] and Chandrakant Tayade[1]

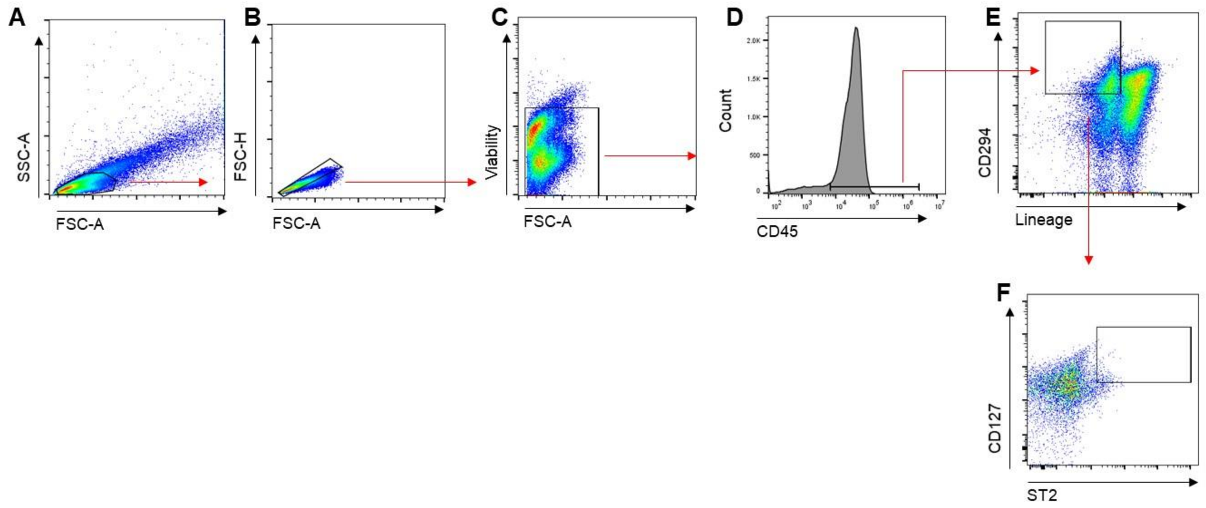

Supplementary Figure 1 Representative full gating strategy for immune profiling conducted on human PF samples to categorize ILC2s. (A) Forward and side scatter gate. (B) Singlet gate. (C) Viability gate set using ½ heat killed and FMO. (D) $CD45^+$ gate. (E) $Lineage^-$ and $CD294^+$ gate. (F) $CD127^+$ and $ST2^+$ gate.

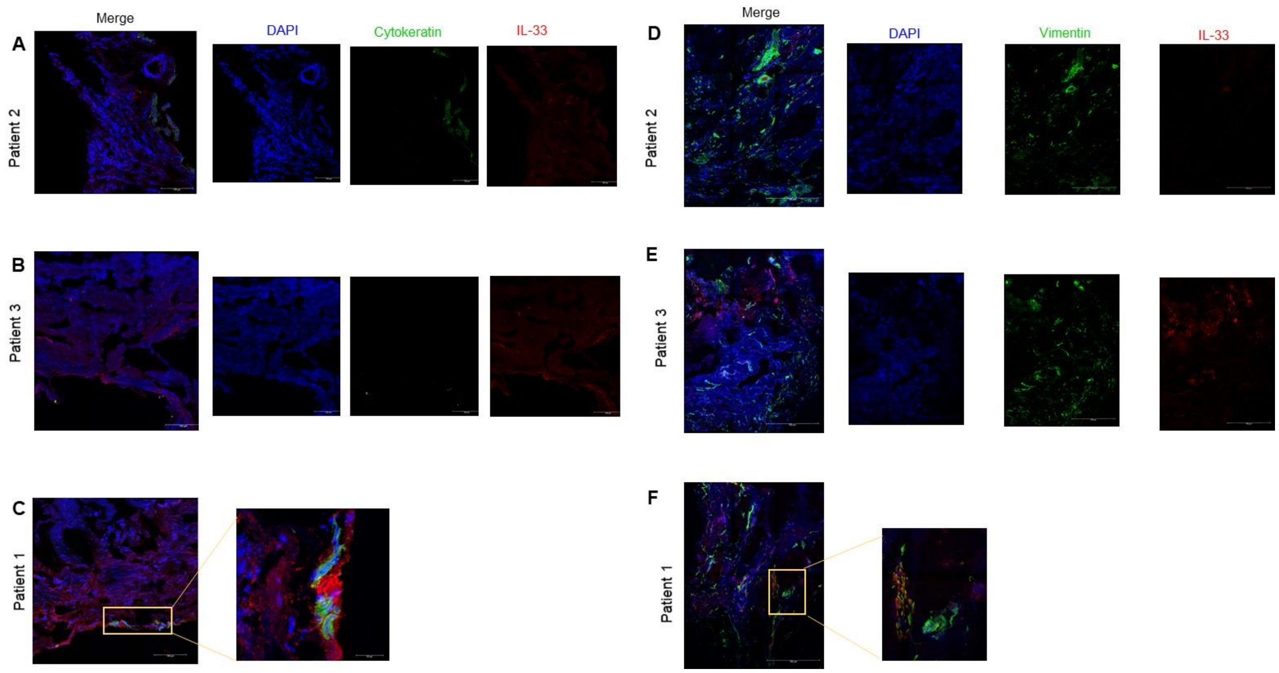

Supplementary Figure 2: IL-33 expression in endometriosis patient samples

Immunofluorescence staining demonstrating IL-33 colocalizes to cytokeratin and vimentin in the endometriotic lesion (n=3). Scale bar 100μm and 20μm.

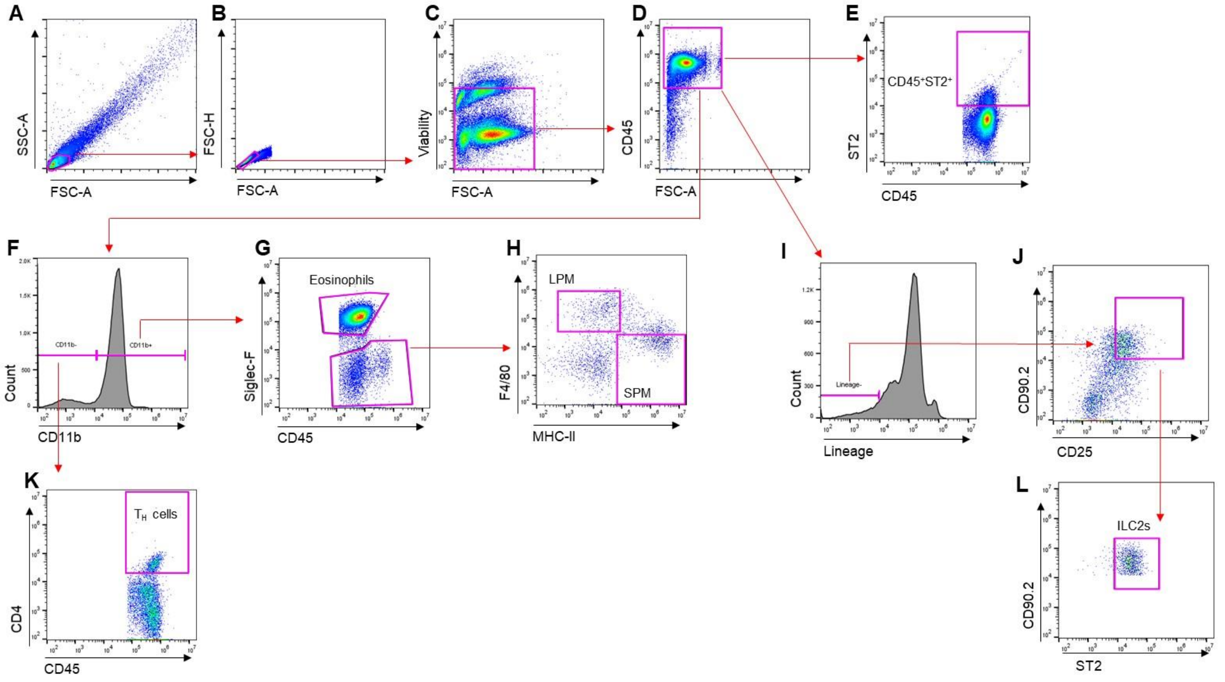

Supplementary Figure 3 Representative full gating strategy for immune profiling conducted on mouse PF samples to categorize CD45$^+$ ST2$^+$ cells, Eosinophils, LPM, SPM, ILC2s and T$_H$ cells. (A) Forward and side scatter gate. (B) Singlet gate. (C) Viability gate set using ½ heat killed and FMO. (D) CD45$^+$ gate. (E) ST2$^+$ gate. (F) CD11b$^+$. (G) Siglec-F$^+$ gate (Eosinophils). (H) F4/80$^+$ and MCH-II$^+$ gate (LPM and SPM). (I) Lineage gate (J) CD90.2$^+$ and CD25$^+$ gate. (K). CD4$^+$ gate (T$_H$ cells). (L) CD90.2$^+$ and ST2$^+$ gate (ILC2s).

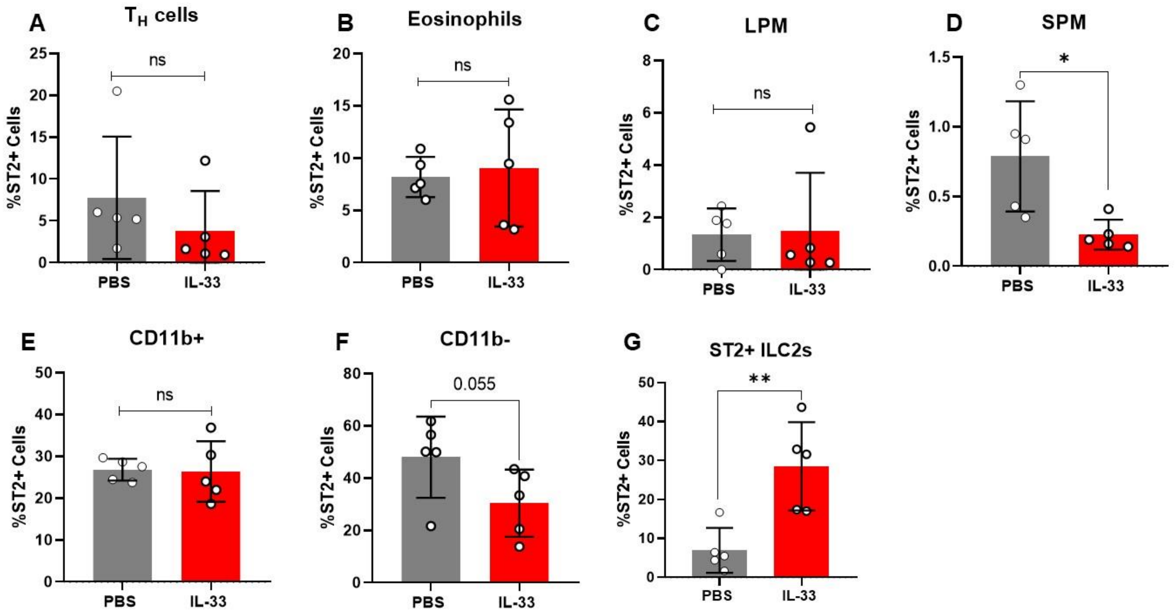

Supplementary Figure 4: Frequency of ST2$^+$ cells in WT endometriosis mice treated with PBS or IL-33. (A) Relative abundance of T_H cells. (B) Relative abundance of Eosinophils. (C) Relative abundance of LPM. (D) Relative abundance of SPM. (E) Relative abundance of CD11b$^+$. (F) Relative abundance of CD11b$^-$. (G) Relative abundance of ILC2s. *P<0.05 **P<0.01 ***P<0.001 ns= Not significant. Mean ±SD are shown. Non-parametric student's t-test with Mann-Whitney.

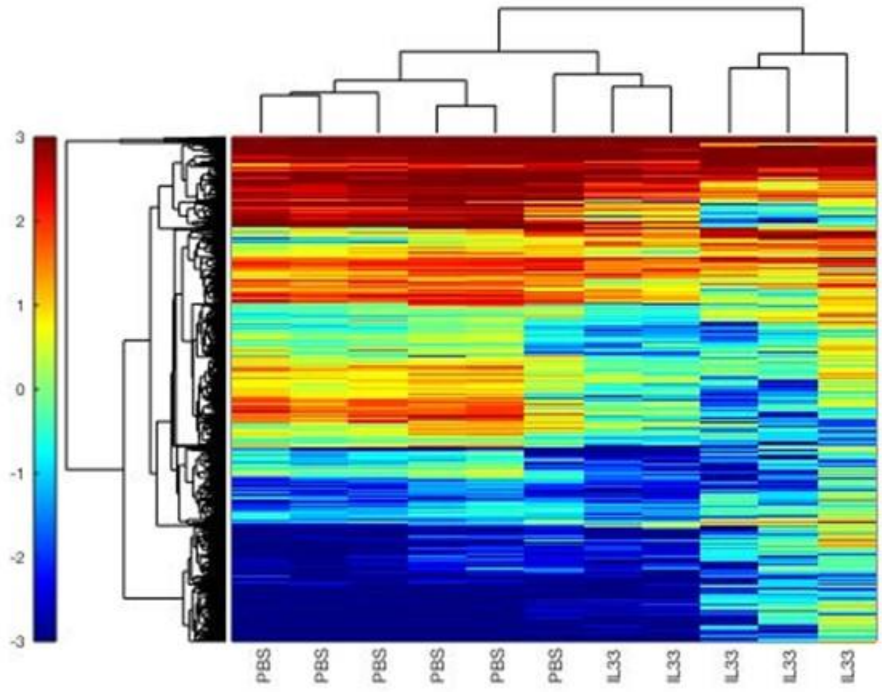

Supplementary Figure 5: Unsupervised hierarchical analysis of murine lesion RNA gene expression raw counts to check for outliers.

Supplementary Tables

Interleukin-33 activates group 2 innate lymphoid cell expansion and modulates endometriosis

Jessica E. Miller[1], Harshavardhan Lingegowda[1], Lindsey K. Symons[1], Olga Bougie[2], Steven L. Young[3], Bruce A. Lessey[4], Madhuri Koti[1,2] and Chandrakant Tayade[1]

Supplementary Table 1: Differentially expressed genes in the WT IL-33 treated lesions compared to WT PBS treated lesions.

Probe Name	Differential Expression Ratio of IL33 vs. PBS	P value of: IL33 vs. PBS	Lower 95% CI of: IL33 vs. PBS	Upper 95% CI of: IL33 vs. PBS	FDR adjusted pvalue of: IL33 vs. PBS
Abca1	-3.49	0.01227255	0.12	-1.45	0.34
Abcb11	11.64	0.00802333	2.28	59.53	0.29
Acaa2	4.45	0.01154738	1.56	12.7	0.33
Acox2	11.04	0.00256852	3.03	40.2	0.21
Acsm3	5.33	0.03307723	1.14	24.82	0.58
Acta2	-4.13	0.00014159	0.14	-2.46	0.1
Adam9	3.66	0.01012799	1.53	8.8	0.32
Adh1	-6.29	0.00861293	0.05	-1.79	0.3
Adh4	6.77	0.01879833	1.46	31.3	0.4
Adh6a	5.82	0.01605164	1.55	21.92	0.37
Aim2	2.24	0.04535111	1	5.03	0.7
Akt1	1.21	0.04356436	1	1.45	0.69
Alad	2.33	0.02739235	1.13	4.79	0.52
Aldob	7.81	0.0051314	2.15	28.36	0.26
Amotl1	-1.47	0.01836705	0.5	-1.09	0.4
Ap1g1	2.74	0.01455747	1.29	5.81	0.35
Apcs	7.6	0.00194525	2.71	21.31	0.19
Aplp2	-1.9	0.03442447	0.3	-1.06	0.59
Apoa1	19.36	0.00138435	4.87	76.98	0.17
Apoa2	10.48	0.00661695	2.77	39.56	0.26
Apoa4	7.94	0.01281973	1.8	35.11	0.34

Apob	11.96	0.00554991	3.17	45.15	0.26
Apoc2	8.75	0.01238698	1.93	39.62	0.34
Apoc3	16.03	0.0065985	2.95	87.17	0.26
Apoh	8.68	0.00229335	2.73	27.63	0.2
Aqp8	9.16	0.00475492	2.6	32.33	0.25
Arhgap35	-4.16	0.00034818	0.13	-2.31	0.12
Atf4	11.46	0.00291591	3.29	39.85	0.22
Atf7ip	-2.67	0.00517932	0.21	-1.5	0.26
Atg101	2.68	0.04523357	0.98	7.37	0.7
Atox1	-2.26	0.0231993	0.22	-1.14	0.46
Atp6v1e1	-1.69	0.00733258	0.43	-1.23	0.28
Atp6v1f	-2.52	0.00867357	0.22	-1.38	0.3
Atp7a	-2	0.01578183	0.3	-1.19	0.37
Axl	-1.84	0.0188597	0.34	-1.14	0.4
Banf1	-1.69	0.04036224	0.36	-1.03	0.65
Bcap31	-2.42	0.0077054	0.24	-1.37	0.28
Bcl2	1.94	0.05021782	0.98	3.85	0.74
Bcl2l1	-2.08	0.01612361	0.27	-1.19	0.37
Blk	4.35	0.04372309	1.03	18.35	0.69
Bmp10	5.08	0.03906703	1.04	24.87	0.64
Bpi	6.69	0.01120998	1.71	26.19	0.33
Bst2	-1.64	0.00054125	0.5	-1.33	0.13
C1s1	-6.38	0.01237207	0.04	-1.69	0.34
C3ar1	9.89	0.00037161	3.82	25.61	0.12
C6	7.89	0.0347354	1.15	54.24	0.6
C8a	6.34	0.00897971	1.79	22.44	0.3
C8b	10.76	0.00418185	2.78	41.65	0.24
C9	6	0.03011171	1.26	28.65	0.54

Calm1	-3.31	0.03553678	0.1	-1.11	0.61
Card9	4.46	0.00843299	1.65	12.07	0.3
Casp4	-2.59	0.00757249	0.21	-1.4	0.28
Casp8	-2.52	0.01168966	0.21	-1.31	0.33
Cbl	-1.59	0.02964163	0.42	-1.05	0.54
Ccr1	2.71	0.02037522	1.21	6.08	0.42
Ccr4	6.01	0.00147148	2.45	14.78	0.17
Cd163	8.87	0.0000861	4.58	17.17	0.1
Cd19	4.5	0.01362066	1.46	13.85	0.34
Cd209e	7.58	0.01281162	1.73	33.13	0.34
Cd34	-4.09	0.01219473	0.09	-1.45	0.34
Cd3d	4.56	0.0474469	1.02	20.44	0.72
Cd3g	5.03	0.00922377	1.67	15.16	0.31
Cd4	3.63	0.03187909	1.14	11.57	0.57
Cd8a	9.39	0.00568551	2.39	36.84	0.26
Cd8b1	12.4	0.00161468	3.47	44.38	0.17
Cdk4	-3.39	0.00225826	0.17	-1.96	0.2
Cdkn2c	-2.13	0.00558182	0.29	-1.34	0.26
Ceacam3	18.33	0.00144761	5.59	60.1	0.17
Cebpa	2.48	0.01915353	1.2	5.12	0.41
Cfhr1	9.18	0.01334952	1.79	47.03	0.34
Cfhr2	-7.17	0.00410687	0.05	-2.65	0.24
Cflar	-3.03	0.01551171	0.14	-1.33	0.37
Chmp4b	2.22	0.0203224	1.17	4.21	0.42
Col10a1	9.6	0.00424453	2.67	34.55	0.24
Col14a1	-4.87	0.00097588	0.1	-2.4	0.15
Col16a1	-4.29	0.00074467	0.13	-2.31	0.13
Col1a1	-4.34	0.01426821	0.08	-1.47	0.35

Col3a1	-49.83	0.00347505	0	-7.17	0.23
Col4a2	-5.68	0.00051128	0.09	-3	0.13
Col5a1	-5.15	0.00007233	0.11	-2.99	0.1
Col6a3	-5.74	0.00023567	0.1	-3.15	0.12
Col6a5	4.58	0.00833465	1.69	12.36	0.29
Col7a1	-2.14	0.01229848	0.27	-1.23	0.34
Cox6a1	-1.39	0.02980497	0.54	-1.04	0.54
Cox7c	2.16	0.02928135	1.09	4.31	0.54
Cpb2	12.8	0.00065579	4.06	40.42	0.13
Creb3	4.08	0.01073234	1.55	10.78	0.32
Crebbp	-1.97	0.01841591	0.3	-1.17	0.4
Csnk1a1	-5.63	0.00057765	0.08	-2.62	0.13
Csnk1g3	2.62	0.02055491	1.2	5.68	0.42
Csnk2b	-2.31	0.03769501	0.2	-1.05	0.63
Ctbp2	-3.13	0.0130579	0.14	-1.41	0.34
Ctnnb1	-1.94	0.03007834	0.29	-1.1	0.54
Ctnnd1	-1.81	0.00048182	0.44	-1.44	0.13
Ctsb	-14.34	0.02094215	0.01	-1.78	0.43
Cul3	-2.6	0.0033729	0.22	-1.5	0.23
Cxcl11	12.71	0.00280718	3.2	50.38	0.22
Cxcl12	-5.52	0.005786	0.06	-1.94	0.26
Cxcr1	7.12	0.03637734	1.1	46.1	0.62
Cybb	-6.91	0.01044089	0.04	-1.87	0.32
Cycs	4.29	0.04576806	0.93	19.77	0.7
Cyfip1	-2.8	0.02105385	0.16	-1.26	0.43
Cyp1a1	11.39	0.00530291	2.68	48.45	0.26
Cyp1a2	8.3	0.00979958	1.91	35.94	0.32
Cyp2c29	12.26	0.00163445	3.62	41.52	0.17

Cyp2c65	8.95	0.01955155	1.63	49.22	0.41
Cyp2j6	6.6	0.00746279	1.98	22.07	0.28
Cyp4a10/31/32	16.34	0.00296695	3.91	68.18	0.22
Cyp4a12a	10.26	0.00728073	2.38	44.35	0.28
Cyp7a1	18.15	0.00549851	3.24	101.7	0.26
Cyp8b1	15.76	0.00377785	3.25	76.32	0.23
Dll1	2.59	0.03984625	1.06	6.36	0.65
Dock1	-4.18	0.02361039	0.08	-1.32	0.47
Dusp7	-1.42	0.0317735	0.52	-1.04	0.57
Dusp8	1.99	0.04002929	1.03	3.85	0.65
Dynll1	4.09	0.00764715	1.61	10.41	0.28
E2f4	1.51	0.04855892	0.99	2.3	0.72
Eef2k	1.57	0.02222591	1.1	2.24	0.45
Eln	-7.42	0.01621553	0.03	-1.63	0.37
Eomes	6.53	0.02346337	1.4	30.52	0.47
Epas1	-1.95	0.00106565	0.38	-1.45	0.16
Ephx2	4.67	0.03557989	1.13	19.32	0.61
Erc1	4.43	0.02996568	1.18	16.56	0.54
Ero1l	7.34	0.00000088	5.09	10.58	0
Erp29	-4.67	0.00124681	0.11	-2.38	0.17
F5	3	0.04032313	1.05	8.54	0.65
F8	3.75	0.01727549	1.36	10.37	0.38
Fap	-3.55	0.00728439	0.13	-1.59	0.28
Fbp1	9.22	0.00090996	3.21	26.51	0.14
Fcer1a	5.26	0.01726038	1.47	18.78	0.38
Fcrlb	7.65	0.00012896	3.89	15.07	0.1
Fga	11.34	0.01291506	2.02	63.63	0.34
Fgb	9	0.00973132	2.04	39.78	0.32

Fgf15	6.72	0.01729985	1.56	28.87	0.38
Fgf2	4.53	0.04180442	1.06	19.42	0.66
Fgf21	12.47	0.00397181	2.99	52.02	0.23
Fgg	9.58	0.01879797	1.67	54.88	0.4
Fli1	-2.29	0.00064658	0.3	-1.6	0.13
Flnb	-2.23	0.00123972	0.32	-1.59	0.17
Flt4	-2.25	0.04814677	0.19	1.03	0.72
Fpr1	5.61	0.00607663	1.88	16.72	0.26
Gas1	-11.11	0.00087074	0.04	-4.36	0.14
Gata3	5.91	0.00024094	3.01	11.62	0.12
Gcnt1	3.55	0.00126843	2.03	6.22	0.17
Gnb4	3.32	0.01849949	1.32	8.37	0.4
Gnptab	-1.87	0.04793775	0.29	-1	0.72
Got2	2.1	0.03685704	1.07	4.13	0.62
Gpc4	-2.61	0.00478852	0.23	-1.53	0.25
Gpr65	2.12	0.01814704	1.19	3.78	0.4
Gpx3	10.12	0.00061438	3.82	26.83	0.13
Grb10	-2.31	0.02001477	0.22	-1.18	0.42
Gzma	4.05	0.01170056	1.48	11.07	0.33
Gzmb	3.21	0.02454224	1.21	8.57	0.48
Gzmd	6.55	0.00791033	1.86	23.03	0.29
Gzme	7.6	0.00548224	2.39	24.18	0.26
H2-Aa	-3.21	0.00881477	0.14	-1.49	0.3
H2-D1	-8.14	0.00582634	0.04	-2.42	0.26
H2-DMb2	3.59	0.01959618	1.28	10.13	0.41
H2-Pa	18.52	0.00367747	3.62	94.65	0.23
H2-Q1	7.11	0.01272079	1.71	29.49	0.34
H2-Q2	6.73	0.00600666	2.17	20.85	0.26

Hadh	5.45	0.02816701	1.31	22.76	0.53
Hadha	-3.59	0.00864818	0.13	-1.66	0.3
Havcr1	10.01	0.01217596	1.91	52.36	0.34
Hc	15.84	0.00324869	3.55	70.63	0.23
Hcfc1	-2.73	0.04659065	0.13	1.02	0.71
Hif1a	-3.11	0.02671925	0.13	-1.23	0.51
Hikeshi	2.07	0.02962079	1.1	3.89	0.54
Hkdc1	15.25	0.00497559	3.02	76.99	0.25
Hmgcs2	4.76	0.02906176	1.2	18.85	0.54
Hsbp1	-3.52	0.04756213	0.08	-1.02	0.72
Hsd11b1	5.33	0.00098667	2.44	11.64	0.15
Hsp90aa1	-4.54	0.00170906	0.11	-2.19	0.18
Hsp90ab1	-12.06	0.01316405	0.01	-2.06	0.34
Hspg2	-3.39	0.00039	0.18	-2.12	0.12
Htra2	3.03	0.0111293	1.37	6.67	0.33
Ifng	13.46	0.00051557	4.63	39.17	0.13
Igf1r	2.73	0.03202733	1.1	6.76	0.57
Ihh	8.76	0.0362536	1.17	65.66	0.61
Ikbkb	-3.06	0.01071936	0.15	-1.42	0.32
Il10	5.53	0.00812566	1.78	17.16	0.29
Il10ra	2.81	0.01096204	1.35	5.87	0.33
Il11	10.74	0.00490976	2.71	42.65	0.25
Il12rb2	7.62	0.04083721	0.99	58.59	0.65
Il13	13.41	0.00147749	3.81	47.13	0.17
Il17a	9.92	0.01232608	1.91	51.59	0.34
Il27	14.61	0.00328991	3.18	67.03	0.23
Il2ra	3.08	0.02433721	1.2	7.87	0.48
Il2rb	7.36	0.00434242	2.38	22.76	0.24

Il4	8.6	0.00312868	2.7	27.38	0.23
Il6st	1.74	0.03254509	1.04	2.9	0.58
Irf4	3.2	0.03509893	1.1	9.28	0.6
Isg15	-2.85	0.00489874	0.19	-1.54	0.25
Itga1	-1.6	0.01004328	0.45	-1.15	0.32
Itgb1	-1.94	0.0248495	0.3	-1.13	0.49
Jag2	5.82	0.00530849	2.04	16.58	0.26
Jak1	-4.85	0.03757164	0.05	-1.12	0.63
Jak2	-4.6	0.00725345	0.08	-1.71	0.28
Kansl1	-2.11	0.03274585	0.24	-1.07	0.58
Kat6a	-1.66	0.01430496	0.41	-1.14	0.35
Kdm2a	-3.9	0.01358974	0.1	-1.45	0.34
Kdm3b	8.75	0.00223926	2.76	27.73	0.2
Kdm5d	10.15	0.0061697	2.43	42.42	0.26
Kdr	-4.02	0.01002556	0.1	-1.56	0.32
Kir3dl1/2	6.6	0.00261071	2.42	18.02	0.21
Klkb1	8.58	0.0062477	2.32	31.75	0.26
Klrb1	7.3	0.00351341	2.3	23.19	0.23
Kng1	6.77	0.0219161	1.39	33.04	0.44
Lama3	2.36	0.03322103	1.09	5.13	0.58
Lamc1	-2.21	0.02507008	0.23	-1.15	0.49
Lamtor2	1.57	0.04017283	1.02	2.42	0.65
Lamtor4	11.28	0.00459197	2.9	43.82	0.25
Lats2	-3.9	0.02595519	0.08	-1.22	0.5
Lep	5.69	0.00929706	1.71	18.96	0.31
Lgals3	2.71	0.01839929	1.22	5.99	0.4
Lipa	-3.92	0.01536246	0.09	-1.44	0.37
Lipc	13.16	0.002492	3.46	50.14	0.21

Lipg	2.17	0.01433121	1.21	3.89	0.35
Lox	-2.57	0.01003406	0.2	-1.35	0.32
Loxl1	-4.22	0.00276836	0.1	-1.87	0.22
Loxl2	-5.43	0.00002217	0.11	-3.38	0.06
Lrp1	-2.48	0.0408415	0.17	-1.03	0.65
Lta	10.33	0.01382425	1.97	54.25	0.34
Lyn	-1.51	0.02857377	0.46	-1.05	0.54
Maml1	1.92	0.00552331	1.28	2.88	0.26
Map1lc3a	4.63	0.00595363	1.82	11.72	0.26
Mapk10	8.02	0.01607716	1.62	39.59	0.37
Marco	6	0.01315996	1.61	22.33	0.34
Masp1	2.53	0.03743764	1.05	6.07	0.63
Mecp2	1.42	0.00518082	1.14	1.76	0.26
Mfap3	-1.85	0.00035316	0.43	-1.46	0.12
Mki67	-2.84	0.00429685	0.19	-1.51	0.24
Mlxipl	5.66	0.01167704	1.65	19.38	0.33
Mmp10	5.57	0.04006653	1.09	28.45	0.65
Mmp13	7.32	0.04145312	1.06	50.7	0.66
Mmp14	-2.44	0.00616743	0.23	-1.4	0.26
Mmp1a	5.93	0.00595907	2.04	17.23	0.26
Mmp1b	13.27	0.0042225	3.24	54.3	0.24
Mmp2	-5.3	0.00992755	0.06	-1.7	0.32
Mmp8	3.4	0.01620556	1.32	8.77	0.37
Mmut	-1.69	0.01976634	0.39	-1.12	0.41
Ms4a1	4.37	0.04374777	1.04	18.32	0.69
Ms4a2	18.18	0.00317033	3.58	92.37	0.23
Mtmr4	-1.73	0.0301222	0.35	-1.06	0.54
Muc5b	12.35	0.01232713	1.96	77.68	0.34

Mylk	-7.26	0.00143971	0.06	-2.94	0.17
Ncam1	-2.43	0.03394944	0.18	-1.09	0.59
Ncf1	3.5	0.00034183	2.09	5.85	0.12
Ncor2	1.53	0.0291071	1.05	2.24	0.54
Ncr1	3.58	0.04561267	1.01	12.69	0.7
Ndufa1	1.93	0.01621247	1.16	3.2	0.37
Ndufc1	-1.82	0.0007713	0.42	-1.39	0.13
Ndufs3	-2.36	0.01105494	0.23	-1.28	0.33
Nedd8	1.36	0.01340244	1.08	1.72	0.34
Nfkb1	-1.81	0.0135242	0.36	-1.17	0.34
Nid1	-2.33	0.0144082	0.23	-1.24	0.35
Nlrp3	6.76	0.00164852	2.5	18.27	0.17
Nol7	7.67	0.00559885	2.3	25.52	0.26
Nos1	8.84	0.0163549	1.61	48.45	0.37
Notch2	-2.38	0.02168725	0.21	-1.19	0.44
Npc1	1.85	0.0095595	1.22	2.83	0.31
Nphp4	4.55	0.01658177	1.42	14.63	0.37
Nr1h3	2.65	0.01373545	1.27	5.54	0.34
Nr1h4	10.59	0.00677102	2.35	47.67	0.27
Nrip3	11.95	0.01197677	2.15	66.55	0.34
Ogt	-2.48	0.01752453	0.2	-1.24	0.39
P3h3	-2.01	0.00062616	0.38	-1.52	0.13
P4hb	-1.88	0.00240567	0.38	-1.35	0.21
Pccb	-1.72	0.01629607	0.38	-1.13	0.37
Pck1	4.5	0.01597093	1.42	14.29	0.37
Pde2a	4.55	0.00121125	2.16	9.58	0.17
Pde3b	2.74	0.00012119	1.92	3.9	0.1
Pdgfrb	-2.08	0.04063229	0.24	-1.03	0.65

Pecam1	-3.86	0.02987663	0.08	-1.17	0.54
Pgk1	4.03	0.00056062	2.24	7.25	0.13
Pgm2	3.52	0.00380375	1.69	7.33	0.23
Phlpp1	-1.45	0.04420559	0.48	-1.01	0.69
Pidd1	3.79	0.0255864	1.19	12.04	0.5
Pik3ca	-2.19	0.00516013	0.28	-1.37	0.26
Pik3cb	2.66	0.01076039	1.35	5.25	0.32
Plcg2	-2.19	0.03425663	0.22	-1.07	0.59
Plg	15.7	0.00145495	4.13	59.72	0.17
Plpp4	13.5	0.0150779	1.96	92.79	0.36
Pnoc	9.89	0.00362588	2.96	33.06	0.23
Pnpla3	8.07	0.00367888	2.41	27.02	0.23
Ppara	3.92	0.03565761	1.07	14.37	0.61
Pparg	3.76	0.04831184	1.01	14.05	0.72
Ppp2ca	-4.53	0.00246687	0.1	-2.01	0.21
Prf1	3.28	0.03777893	1.1	9.84	0.63
Prkaa1	-1.64	0.0039361	0.46	-1.23	0.23
Prkaca	-2.39	0.02969261	0.19	-1.07	0.54
Prkag1	3.83	0.01242603	1.44	10.15	0.34
Pros1	-3.19	0.00612177	0.15	-1.57	0.26
Psmb7	-3.68	0.00090229	0.16	-2.12	0.14
Psmb8	-3.35	0.03947135	0.1	-1.07	0.65
Psmc3	-2.28	0.00197604	0.29	-1.51	0.19
Ptk2b	1.38	0.04430066	1.01	1.89	0.69
Ptpa	4.54	0.00308282	2.05	10.04	0.23
Ptpn12	-1.67	0.01070431	0.42	-1.17	0.32
Rac1	-3.23	0.04172651	0.1	-1.07	0.66
Rac2	2.04	0.01310351	1.2	3.46	0.34

Rap1b	-8.64	0.00833333	0.03	-2.27	0.29
Rasgrp4	2.79	0.01385933	1.29	6.02	0.34
Rgs7	5.37	0.01163436	1.68	17.12	0.33
Rho	6.29	0.00276462	2.46	16.12	0.22
Ripk3	2.42	0.00788297	1.35	4.33	0.29
Rnf152	2.65	0.04600161	0.96	7.29	0.7
Rock2	-2.23	0.01084274	0.26	-1.28	0.32
Rora	4.22	0.00068885	2.24	7.91	0.13
Rplp0	-3.13	0.01165131	0.14	-1.41	0.33
Rps27a	5.19	0.01312818	1.56	17.32	0.34
Rps6ka2	2.26	0.00206085	1.48	3.47	0.19
S100a4	2.53	0.00630537	1.39	4.61	0.26
Saa1/2	5.37	0.02453777	1.3	22.2	0.48
Scd2	-7.41	0.03926549	0.02	-1.09	0.65
Scin	7.18	0.00621604	2.12	24.35	0.26
Sdc3	-2.03	0.00592843	0.32	-1.32	0.26
Sec61b	-1.63	0.00025501	0.52	-1.39	0.12
Sem1	1.37	0.02662696	1.05	1.81	0.51
Serpinh1	-13.5	0.00020902	0.03	-5.48	0.12
Sh2d1a	6.51	0.04886186	0.89	47.85	0.72
Siglecf	2.59	0.02048083	1.19	5.61	0.42
Sirt1	1.98	0.0131095	1.21	3.23	0.34
Skp1a	-3.54	0.00158732	0.15	-1.92	0.17
Slc2a2	10.5	0.00708263	2.36	46.79	0.28
Smad4	-2.68	0.03804539	0.15	-1.08	0.63
Snai2	-2.45	0.0495615	0.17	1	0.73
Sod1	-8.11	0.00825016	0.03	-2.28	0.29
Sorbs1	-2.86	0.02095723	0.15	-1.26	0.43

Sp3	-1.79	0.00142834	0.42	-1.34	0.17
Spop	2.67	0.00430429	1.5	4.76	0.24
Spp1	-4.18	0.04677724	0.06	-1.01	0.71
Src	-2	0.03256293	0.26	-1.06	0.58
Ssr2	-5.71	0.00394365	0.07	-2.24	0.23
Ssr4	-2.26	0.02629433	0.22	-1.14	0.51
Stat1	-2.52	0.00184195	0.25	-1.57	0.19
Stk4	-1.69	0.003841	0.44	-1.25	0.23
Sufu	-1.46	0.02760752	0.49	-1.05	0.52
Sugt1	-3.18	0.00924345	0.15	-1.51	0.31
Tbl1xr1	1.91	0.00210039	1.36	2.7	0.19
Tbx21	9.62	0.00713964	2.55	36.34	0.28
Tcl1	17.81	0.00992862	2.81	112.92	0.32
Tgfb1i1	-4.57	0.00855086	0.08	-1.7	0.3
Tgfbr1	-3.42	0.03709276	0.09	-1.08	0.62
Tgfbr2	-5.71	0.00015078	0.1	-3.1	0.1
Thbs2	-4.42	0.04476223	0.05	-1.05	0.7
Thbs3	-2.05	0.00768511	0.31	-1.29	0.28
Timp1	2.58	0.04349058	1.01	6.58	0.69
Timp2	-1.88	0.01376025	0.33	-1.17	0.34
Tjp2	-1.72	0.02144949	0.38	-1.11	0.44
Tln1	-3.57	0.00599223	0.14	-1.73	0.26
Tnf	1.86	0.04708195	1.01	3.45	0.71
Tnfrsf17	7.24	0.00631679	2.03	25.83	0.26
Tnfsf14	2.83	0.00480567	1.49	5.38	0.25
Tnn	3.65	0.01656551	1.33	10.02	0.37
Tpsab1	7.22	0.00364011	2.29	22.78	0.23
Tpsb2	3.34	0.04622694	1.03	10.84	0.71

Tradd	2.91	0.01474387	1.33	6.36	0.35
Trat1	6.62	0.00263467	2.44	17.96	0.21
Trib3	10.65	0.00030224	4.13	27.48	0.12
Txn1	-6.05	0.02762429	0.04	-1.3	0.52
Txn2	-2.44	0.00190593	0.26	-1.57	0.19
Txndc5	-7.57	0.01839511	0.03	-1.66	0.4
Uba52	-6.22	0.01013549	0.05	-1.79	0.32
Ube2d2a	-1.56	0.02944683	0.43	-1.04	0.54
Ube2n	-2.4	0.04821357	0.17	1.02	0.72
Ube4b	-2.36	0.02739406	0.21	-1.15	0.52
Uqcrfs1	2.97	0.02233532	1.24	7.15	0.45
Uqcrh	-2.22	0.01401485	0.26	-1.26	0.34
Vamp8	5.61	0.00345277	2.24	14.07	0.23
Vim	-2.84	0.0038121	0.19	-1.52	0.23
Xbp1	-3.57	0.00325001	0.14	-1.8	0.23
Xcl1	2.94	0.03335825	1.06	8.11	0.58
Xiap	-1.95	0.01666299	0.31	-1.17	0.37
Ywhae	-4.23	0.01070804	0.09	-1.63	0.32
Ywhag	-2.69	0.0103296	0.19	-1.35	0.32
Ywhaq	-4.51	0.00054745	0.13	-2.58	0.13
